# Streamlining data recording through optical character recognition: a prospective multi-center study in intensive care units

**DOI:** 10.1186/s13054-025-05347-1

**Published:** 2025-03-18

**Authors:** Prompak Nitayavardhana, Keibun Liu, Kiyomitsu Fukaguchi, Mineto Fujisawa, Itaru Koike, Aina Tominaga, Yuta Iwamoto, Tadahiro Goto, Jacky Y. Suen, John F. Fraser, Pauline Yeung Ng

**Affiliations:** 1 Division of Cardiothoracic Surgery, Department of Surgery, Faculty of Medicine, Siriraj Hospital, Bangkok, Thailand; 2 Critical Care Research Group, The Prince Charles Hospital, Brisbane, Australia; 3 Institute for Molecular Bioscience, University of Queensland, Brisbane, Australia; 4 Non-Profit Organization, ICU Collaboration Network (ICON), Tokyo, Japan; 5 Department of Emergency Medicine, Shonan Kamakura General Hospital, Kanagawa, Japan; 6 TXP Research, TXP Medical Co. Ltd., Tokyo, Japan; 7 Adult Intensive Care Services, The Prince Charles Hospital, Brisbane, Australia; 8 Queensland University of Technology, Brisbane, Australia; 9 St. Andrews War Memorial Hospital, Brisbane, Australia; 10 Critical Care Medicine Unit, School of Clinical Medicine, The University of Hong Kong, Pokfulam, Hong Kong SAR China; 11 Department of Adult Intensive Care, Queen Mary Hospital, Pokfulam, Hong Kong SAR China

**Keywords:** Intensive care unit, Mobile applications, Optical character recognition, Data entry, Data registry

## Abstract

**Background:**

The manual entry of data into large patient databases requires significant resources and time. It is possible that a system that is enhanced with the technology of optical character recognition (OCR) can facilitate data entry, reduce data entry errors, and decrease the burden on healthcare personnel.

**Methods:**

This was a prospective multi-center observational study across intensive care units (ICU) in 3 countries. Subjects were critically-ill and required invasive mechanical ventilation and extracorporeal life support. Clinical photos from various medical devices were uploaded using an OCR-enhanced case record form. The degree of data completeness, data accuracy, and time saved in entering data were compared with conventional manual data entry.

**Results:**

The OCR-based system was developed with 868 photos and validated with 469 photos. In independent validation by 8 untrained personnel involving 1018 data points, the overall data completeness was 98.5% (range 98.2–100%), while the overall data accuracy was 96.9% (range 95.3–100%). It significantly reduced data entry time compared to manual entry (mean reduction 43.9% [range 27.0–1.1%]). The average data entry time needed per patient were 3.4 (range 1.2–5.9) minutes with the OCR-based system, compared with 6.0 (range 2.2–8.1) minutes with manual data entry. Users reported high satisfaction with the tool, with an overall recommendation rate of 4.25 ± 1.04 (maximum of 5).

**Conclusion:**

An OCR-based data entry system can effectively and efficiently facilitate data entry into clinical databases, making it a promising tool for future clinical data management. Wider uptake of these systems should be encouraged to better understand their strengths and limitations in both clinical and research settings.

**Supplementary Information:**

The online version contains supplementary material available at 10.1186/s13054-025-05347-1.

## Introduction

Despite advancements in digital technology, manual entry of patient data for both clinical and research purposes continue to be a widespread practice in the healthcare setting. In the intensive care unit (ICU), a wide range of clinical data is generated from various sources, including physiological monitors, hemodynamics monitors, ventilators, extracorporeal life support systems, and laboratory investigations. There is a lack of universal data capturing platforms that can be adapted to include information across devices. The resulting large number of available data variables create a huge burden on time needed to enter data into various patient registries. For example, the MIMIC-IV database, a widely-used critical care database, contains over 40,000 distinct variables with a wide range of clinical data points [1], which is further exaggerated in patients with prolonged or complicated stays. The need to dedicate time to manual data entry in the ICU can significantly impact productivity, particularly in some parts of the world where data entry is largely contributed by frontline clinicians or nursing staff who have competing clinical obligations. In addition, dependence on manual processes often leads to unpredictable delays from resource constraints, as well as inaccuracies from human errors.

Digital technology has seen major breakthrough in applications beyond clinical medicine. The ability to extract context-specific information from unstructured data generates a huge time- and cost-saving potential. Prior research has explored the technology of optical character recognition (OCR) for extracting information from standard medical devices [2]. However, the ICU environment poses additional challenges due to the diversity of monitoring equipment and the dynamic nature of the data involved.

To address these challenges, we developed an OCR-based data entry system in alignment with an existing large international patient registry, with the objective of automating numerical data capture from ICU monitors. The hypothesis was that the OCR-based system has satisfactory performance in data accuracy and significant time-saving effects. We evaluated its performance by comparing it to manual data entry in a prospective multi-center observational study.

## Methods

### Study design and settings

This was a prospective multi-center observational study across three countries: Queen Mary Hospital in Hong Kong, Siriraj Hospital in Thailand, and The Prince Charles Hospital in Australia, during the period from April 2023 to February 2024. This study complied with the Declaration of Helsinki 1975 and its later amendments, and was approved by the Institutional Review Board (IRB) of the University of Hong Kong / Hospital Authority Hong Kong West Cluster (IRB Reference Number: UW 23–159, dated 4th April 2023), the Siriraj IRB (Certificate of Approval Number: Si 470/2024, dated 13th June 2024), and the IRB of the Ministry of Health, Labour and Welfare, Japan (IRB number: 21000041, registration number: TXPREC-021, dated 31st July 2024). Due to the non-invasive nature of the study and the de-identified nature of the data, the need for informed consent was waived.

## Development of OCR-based system

The aim of the OCR-based system was to automatically transcribe numerical data from uploaded photos of ICU devices taken with a smartphone into the case report form (CRF). The targeted ICU devices were as follows: (1) physiological monitor, including vital signs; (2) hemodynamic monitor, including measurements such as cardiac output; (3) mechanical ventilator, (4) extracorporeal membrane oxygen (ECMO) console, and (5) computerized display of laboratory data. The details of device manufacturers and models are shown in Supplemental Table 1.

**Table 1:** Data completeness and accuracy in the evaluation phase

	Site	Number of images uploaded	Number of data points	Data completeness* (%)	Data Accuracy* (%)
Physiological monitor	AU	9	65	96.9	96.9
	HK	8	40	97.5	77.5
	HK	5	30	100.0	100.0
	TH	8	46	95.7	95.7
	All sites	30	181	96.7	92.3
Hemodynamic monitor**	AU	10	80	97.5	95.0
	HK	–	–	–	**–**
	TH	–	–	–	–
	All sites	10	80	97.5	95.0
Mechanical ventilator	AU	8	71	100.0	100.0
	HK	8	96	99.0	97.9
	TH	8	89	100.0	100.0
	All sites	24	256	99.6	99.2
ECMO	AU	8	56	98.2	98.2
	HK	8	64	100.0	95.3
	TH	8	51	100.0	100.0
	All sites	24	171	99.4	97.6
Laboratory data	AU	8	104	100.0	100.0
	HK	8	117	98.3	97.4
	TH	9	109	100.0	98.2
	All Sites	25	330	99.4	98.5

AU, Australia; ECMO, extracorporeal membrane oxygenation; HK, Hong Kong; TH, Thailand

^*^The rate of data completeness was calculated as (“Correct” + “Incorrect”) / (“Undetected” + “Correct” + “Incorrect”). The rate of data accuracy was calculated as “Correct” / (“Undetected” + “Correct” + “Incorrect” + “False Positive”)

^**^A standalone hemodynamic monitor was not used in Hong Kong and Thailand

The OCR system was developed on an AWS EC2 G4dn.xlarge instance, equipped with an Intel Xeon Platinum 8259CL CPU, 16 GB of RAM, and an NVIDIA T4 GPU. This setup ran Amazon Linux 2 as its operating system. The system’s software stack included Python 3.8.5 for general programming, PyTorch 1.12.1 for deep learning tasks, and OpenCV 4.7.0 for image processing operations. A technical description of the development of the OCR system is provided in the Supplemental Methods.

The development of the OCR-based data entry system was implemented in three phases. First, the main CRF including variables of interest, and the format and possible ranges of data was created based on the international ELSO Registry that recorded information about patients who received ECMO care [3]. Across the 3 sites, we identified a total of 4 physiology monitors, 2 hemodynamic monitors, 5 mechanical ventilators, 4 ECMO consoles, and 3 laboratory result displays. For each of the devices, we labelled data with cross referencing to the main CRF (Supplemental Figs. 1, 2, 3, 4 and 5). During data entry, images taken of the ICU devices were uploaded to a server via a dedicated mobile phone application. An OCR system installed on the server automatically reads the mapped data from the uploaded images, and transfers the extracted information to the corresponding variable in the CRF. Details of the target parameters from each of the ICU devices are shown in Supplemental Table 2.

## Validation of OCR-based system

In the second phase, we proceeded with model validation and tuning to optimize the performance of the OCR-based system. During this validation process, we focused on several critical aspects: (1) the quality of the photos, (2) misreadings by the OCR-based system, (3) the accuracy of the OCR-based system, and (4) the underlying mechanisms contributing to suboptimal accuracy. Based on the evaluation of these factors, updates were made to the OCR software to improve its performance.

## Evaluation of OCR-based system

Finally, evaluation of the OCR-based system was conducted on an independent test dataset. At each site, 2 independent researchers inputted data using both the OCR-based as well as manual entry methods. These operators were not informed of the research methodology and were blinded to all subsequent analyses. The quality of the data read by the OCR-based system was compared to manually-reviewed data, which was treated as the “gold standard”, and classified into the following categories:

1. “Undetected”: A numerical value is present on the monitor but not detected by OCR.
2. “Correct”: The OCR-detected numerical value matches the gold standard.
3. “Incorrect”: The OCR-detected numerical value does not match the gold standard.
4. “False Positive”: The OCR detects a numerical value that is not present on the monitor.

The rate of data completeness was calculated as (“Correct” + “Incorrect”) / (“Undetected” + “Correct” + “Incorrect”). The rate of data accuracy was calculated as “Correct” / (“Undetected” + “Correct” + “Incorrect” + “False Positive”).

In order to assess potential time-saving effects of the OCR-based system, all data entry personnel independently entered the same set of data using the OCR-based system, as well as by traditional manual itemized data entry into a spreadsheet, and recorded the total time needed to complete the data entry process.

In addition, a user survey was administered to a focus group including data entry personnel from all 3 sites to collect feedback on the user experience. The survey tool is included in Supplemental Table 3. All analyses were performed using Python version 3.8.5.

## Results

### Development and validation of OCR-based system

**Fig. 1:**
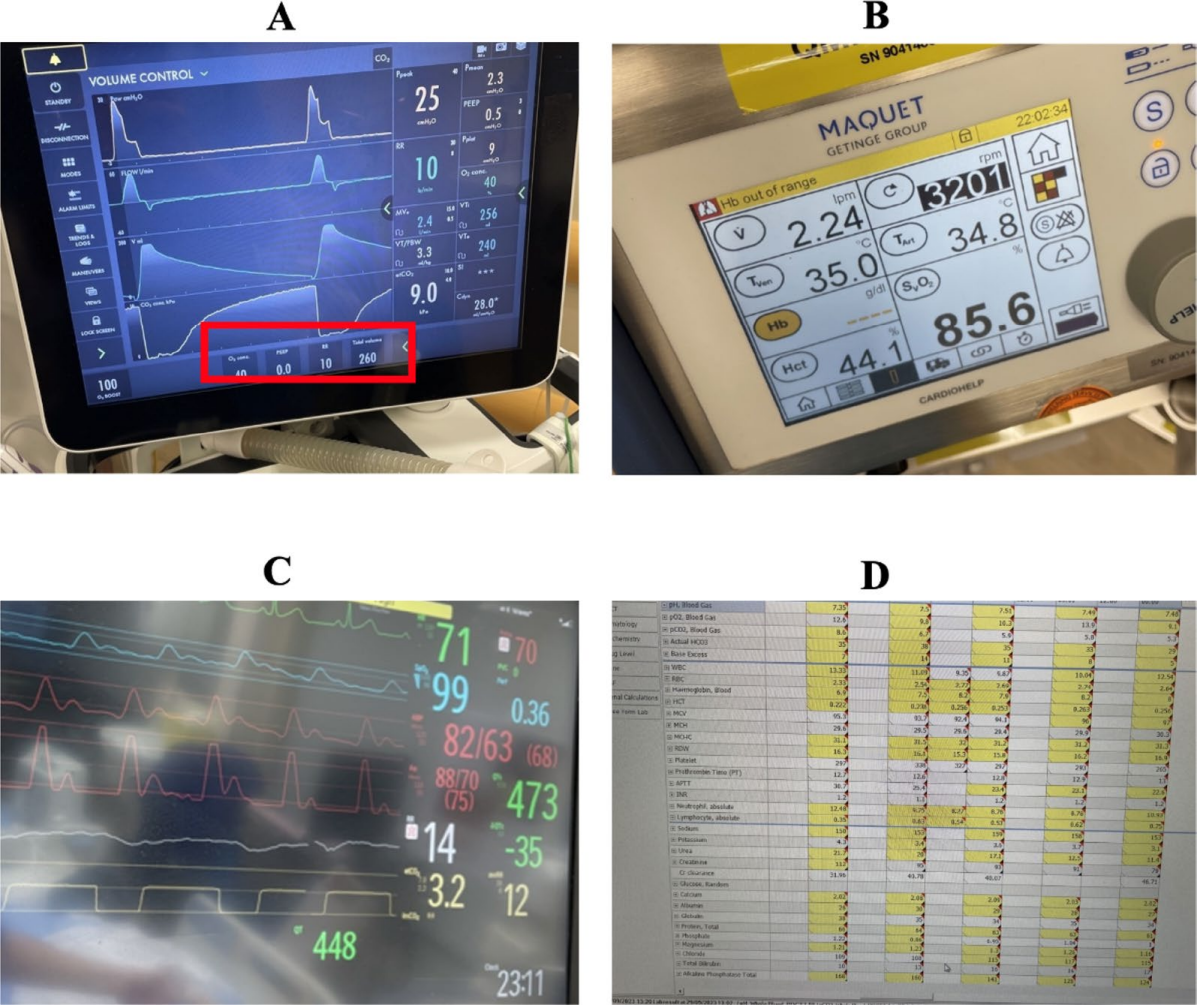
Examples of poor image quality affecting performance of optical character recognition-based system. Examples of poor image quality rendering suboptimal performance of the optical character recognition-based system. These included low image resolution (**A**), photos taken at slanted angles (**B**), presence of strong light reflections (**C**), and parts of the image being cropped out (**D**)

A total of 91 variables were selected and mapped to the OCR-based system. The OCR-based system was developed using a total of 868 photos, averaging 48 photos per device interface (Supplemental Table 1). Deployment of the OCR-based system could be done via a mobile device application or a web-based form. Subsequently, the performance of the software was validated with 469 photos. During the validation process, the most frequent issues rendering suboptimal performance of the OCR-based system were due to poor image quality, for example, low image resolution (Fig. 1A), photos taken at slanted angles (Fig. 1B), presence of strong light reflections (Fig. 1C), and parts of the image being cropped out (Fig. 1D). Subsequent updates were made to the OCR software, including enhancements to improve reading accuracy by training it to recognize variations in the color and size of numbers displayed on the monitor screen.

## Evaluation of OCR-based system

**Fig. 2:**
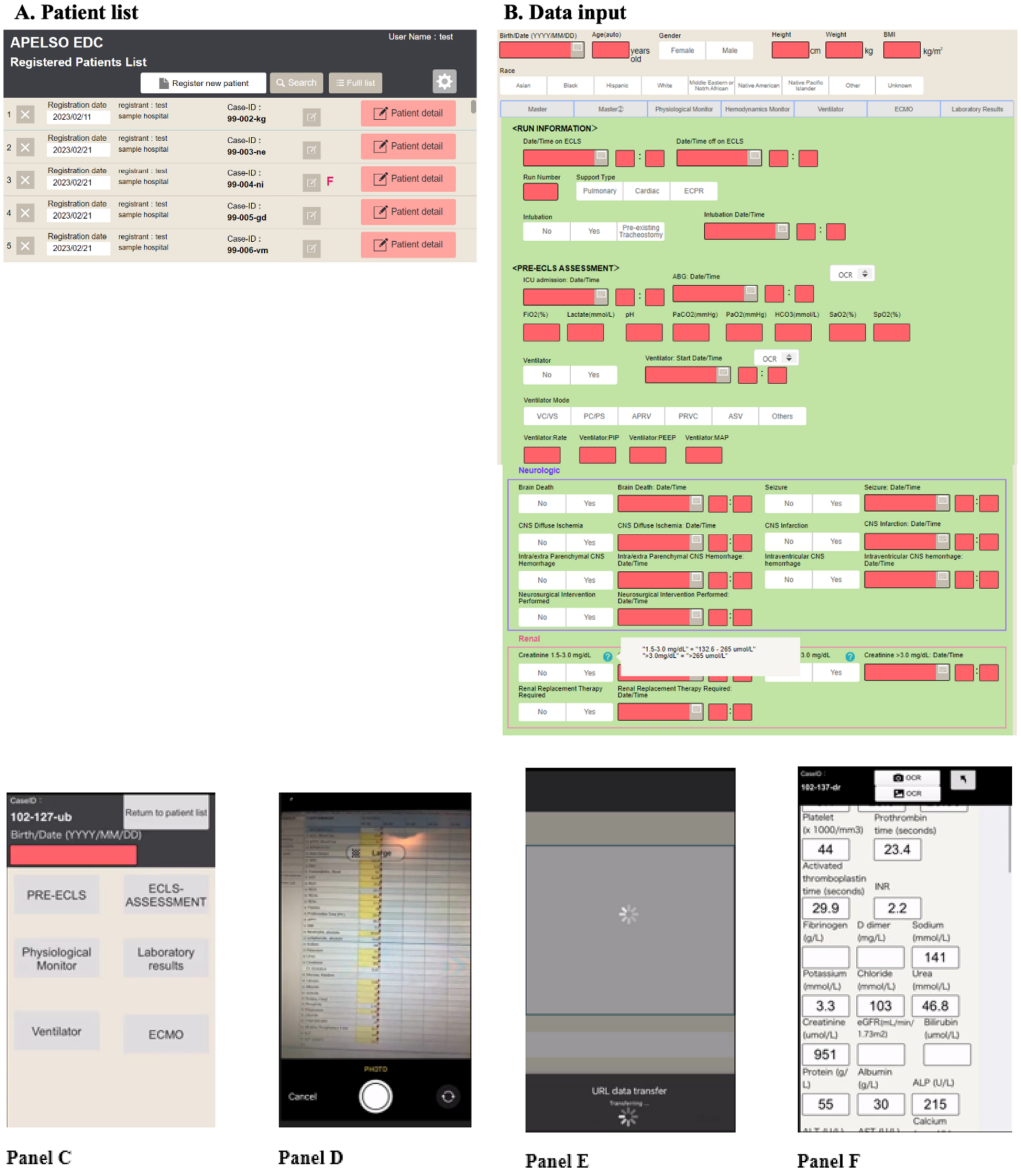
Examples of the user interface of the optical character recognition-based system. The case record form including the patient list (Panel** A**), variables of interest (Panel** B**), and the format and possible ranges of data was created based on the ELSO Registry. The OCR-based system is accessible directly from the case record form (Panel** C**). Subsequently, the camera function is used to capture an image of the target monitor or device (Panel** D**), data is automatically uploaded (Panel **E)**, and relevant parameters are transcribed into the case record form (Panel** F**)

A total of 6 data entry personnel inputted data during the evaluation phase, including 103 photos across 3 sites. Examples of the user interface of the OCR-based system are shown in Fig. 2. The overall data completeness was 98.5%, ranging from 98.2 to 100%, while the overall data accuracy was 96.9%, ranging from 95.3 to 100%. “False Positive” data were not detected, meaning the OCR did not identify any numerical values that were not actually present on the monitors. There was an outlier in the data accuracy for the physiological monitor, which was upon further examination, possibly due to the unexpected display of a second arterial waveform (Supplemental Fig. 2). Detailed results with regards to data completeness and accuracy are presented in Table 1 and Supplemental Table 4.

There were significant time-saving effects noted, with a mean reduction in time needed to complete data entry of 43.9% (range 27.0–81.1%) after using the OCR-based system. The average data entry time needed per patient were 3.4 (range 1.2–5.9) minutes with the OCR-based system, compared with 6.0 (range 2.2–8.1) minutes with manual data entry.

## User satisfaction

A user survey was administered to 8 research assistants. The survey results indicated a generally positive perception of the OCR-based system among users. 7 (87.5%) responders reported “I got used to it right away” for the ease-of-use question. The median usefulness rating for the OCR function was 5 (IQR: 4–5), and the time-saving aspect of the OCR function received a median rating of 5 (IQR: 5–5) (all ratings out of 5). Users also expressed a high median satisfaction rating of 4 (IQR: 3.3–4.8). Overall, the median recommendation rating for the OCR-based system was (IQR: 4–5). Specific issues requiring improvement that were identified by users included the occasional slow performance of the software. Detailed results of the user survey are presented in Supplemental Table 5.

## Discussion

In this prospective pilot study, we provide proof-of-concept data that an OCR-based system can read and record data from ICU monitors into a user-defined CRF with a high degree of data completeness and accuracy. Additionally, we observed substantial time-saving effects and a high level of user satisfaction. There is immense potential for OCR-based data entry to facilitate standardized collection of multiple variables in large datasets, thereby supporting quality improvement, benchmarking, and research efforts, particularly in resource-limited settings.

OCR-based systems have been increasingly utilized in applications to facilitate various human tasks. A common example is their ability to translate text into a preferred language in real time by using mobile device cameras to capture print or photos. This has significantly facilitated multilingual communication by overcoming language barriers between countries. Takano et al. had demonstrated the functionality of automated label data extraction and database generation from herbarium specimens, where handwritten Japanese notes were processed and electronically converting then stored in medical records [4]. Prototypical work in the clinical environment has also been reported, including the extraction of single variables from medication pumps in the neurocritical care setting. [5]

In clinical medicine and research settings, conventional data entry is often characterized by the need to handle a vast number of data variables, which can be overwhelming and error-prone [6, 7]. Studies have shown that the redundancy and inconsistency between different datasets necessitate repeated efforts to ensure data accuracy and completeness before meaningful analyses can be performed and conclusions can be drawn [8, 9]. Especially in situations where numerous registries on similar topics co-exist, this duplication of effort is not only inefficient but also significantly increases the risk of data entry fatigue, and eventual burnout of researchers. The resource-intensive nature of manual data entry practices can be particularly challenging for low-income countries, resulting in obstacles from participation in multicenter registries. For example, data from Asian countries only make up around 10% of ECMO runs captured in the global ELSO registry, which is a departure from actual large patient volumes [10]. In China alone, there was a 38% increase in annual ECMO cases to a total of 3923 cases in 2018 [11]. Similarly, non-European or American patients only represented 28.9% of data in the global ICON audit of ICU admissions [12]. The eventual underrepresentation in clinical research can be partially attributed to the lack of infrastructure and resources needed for systematic data collection and management [13]. The World Health Organization has highlighted that barriers to implementing effective data entry and management systems in low- and middle-income countries further exacerbate health disparities [14]. Even in high-income countries, the rising salary costs for data entry personnel impose a significant burden on financial resources.

While techniques to automate and streamline data input have been developed for certain electronic healthcare record (EHR) systems, these are usually applied to semi-structured data or numerical data that are already recorded. The wealth of information from the clinical environment, such as information from various ICU devices are not considered. These automated systems are also usually implemented in large medical systems in developed countries, and the costs of purchasing user licenses are prohibitive to low- and middle-income countries. [15]

In our study, we showed that an OCR-based system could have the potential to reduce reliance on dedicated data entry personnel while maintaining data quality and enhancing participation in global health research. The significant time-saving effects of > 40% demonstrated in our OCR-based system compared to traditional manual input were paralleled by extremely high user satisfaction. This time-saving effect was also reported by Mohana et al. with 17% reduction in time for data-related tasks while maintaining a near 100% accuracy rate, ultimately reducing the cost of manual document processing by 35% [16]. The ability to capture clinical data at the bedside using readily-available mobile devices allows seamless integration of data collection into clinical workflows. Important time-sensitive clinical events, such as changes in physiology and hemodynamics, together with concurrent ventilator and ECMO readings, can be recorded at the moment of patient deterioration [2]. The high rate of data completeness and data accuracy with the OCR-based system, even among untrained users, obviates the need for dedicated training of data entry personnel and incentivizes more widespread participation from clinical staff. Taken together, an OCR-based approach lowers the data collection barrier by harnessing data that could be contributed from standalone legacy monitors without network connectivity, eliminating the need to understand complex data dictionaries, and serving as a manufacture-agnostic and low-cost approach to data collection.

Near-future modification of these OCR-based systems include the incorporation of multilingual capabilities to circumvent language barriers that are particularly prevalent in low- to middle-income countries. In addition, it is possible that the incorporation of natural language processing capabilities would augment data capture from unstructured clinical notes [17]. The widespread testing and implementation of these OCR- and AI-augmented systems in conjunction with existing international patient registries and across different healthcare settings will provide real-world data on the potential and limitations of this technology, allowing understanding of practical and regularly limitations, and setting a precedent to revolutionize data entry practices for large multicenter studies. Lastly, given that the federated approach to cross-institutional data sharing and analysis has experienced early success [18], more widespread integration of case report forms with federated data platforms should be implemented. As the OCR technology evolves, it may serve as a key component in improving real-world data collection and supporting data-driven advancements in critical care medicine.

## Limitations

Firstly, this is a pilot study of the OCR-based system and more widespread testing across healthcare settings is needed. There is a need to work closely with software developers to adapt the system according to geographic locality, and it remains possible that the system may underperform with certain languages or different types of monitor displays. Secondly, it was difficult to standardize the baseline experience level and data entry practices across different users who participated in the time-saving analysis, lending possible biases in time needed to enter data. Finally, it remains to be assessed whether inaccuracies of the OCR-based system may introduce information biases in clinical studies.

## Conclusions

We developed and validated an OCR-based system that enhances data capture in the ICU with a high degree of data completeness and accuracy. This breakthrough in technology should change the workflow and substantially alleviate the burden of data entry, potentially translating to the advancement of data contribution to international registries and the development of data-driven solutions in the critical care environment.

## Supplementary Information

Additional file 1.

## Data Availability

No datasets were generated or analysed during the current study.

## Abbreviations

CRF: Case report form
ECMO: Extracorporeal membrane oxygenation
EHR: Electronic healthcare record
ELSO: Extracorporeal Life Support Organization
ICU: Intensive care units
IRB: Institutional Review Board
MIMIC-IV: Medical Information Mart for Intensive Care
OCR: Optical character recognition

## References

[1] Johnson AEW, Bulgarelli L, Shen L, et al. MIMIC-IV, a freely accessible electronic health record dataset. Sci Data. 2023;10(1):1. 10.1038/s41597-022-01899-x. 36596836 10.1038/s41597-022-01899-x PMC9810617

[2] Soeno S, Liu K, Watanabe S, Sonoo T, Goto T. Development of novel optical character recognition system to reduce recording time for vital signs and prescriptions: a simulation-based study. PLoS ONE. 2024;19(1): e0296319. 10.1371/journal.pone.0296319. 38241403 10.1371/journal.pone.0296319 PMC10798482

[3] Extracorporeal Life Support Organization (ELSO) Registry. Extracorporeal Life Support Organization. (https://www.elso.org/registry.aspx).

[4] Takano A, Cole TCH, Konagai H. A novel automated label data extraction and data base generation system from herbarium specimen images using OCR and NER. Sci Rep. 2024;14(1):112. 10.1038/s41598-023-50179-0. 38167449 10.1038/s41598-023-50179-0 PMC10761843

[5] Froese L, Dian J, Batson C, Gomez A, Sainbhi AS, Unger B, et al. Computer vision for continuous bedside pharmacological data extraction: a novel application of artificial intelligence for clinical data recording and biomedical research. Frontiers in Big Data. 2021;27:4. 10.3389/fdata.2021.689358. 10.3389/fdata.2021.689358 PMC8430398 34514379

[6] Hogan WR, Wagner MM. Accuracy of data in computer-based patient records. J Am Med Inform Assoc. 1997;4(5):342–55. 10.1136/jamia.1997.0040342. 9292840 10.1136/jamia.1997.0040342 PMC61252

[7] Arts DG, De Keizer NF, Scheffer GJ. Defining and improving data quality in medical registries: a literature review, case study, and generic framework. J Am Med Inform Assoc. 2002;9(6):600–11. 10.1197/jamia.m1087. 12386111 10.1197/jamia.M1087 PMC349377

[8] Kahn MG, Callahan TJ, Barnard J, et al. A harmonized data quality assessment terminology and framework for the secondary use of electronic health record data. EGEMS (Wash DC). 2016;4(1):1244. 10.13063/2327-9214.1244. 27713905 10.13063/2327-9214.1244 PMC5051581

[9] Weiskopf NG, Weng C. Methods and dimensions of electronic health record data quality assessment: enabling reuse for clinical research. J Am Med Inform Assoc. 2013;20(1):144–51. 10.1136/amiajnl-2011-000681. 22733976 10.1136/amiajnl-2011-000681 PMC3555312

[10] ECLS Registry Report International Summary - July 2020. Extracorporeal Life Support Organization. (https://www.elso.org/registry/statistics.aspx).

[11] Li CL, Hou XT, Hei FL, et al. China statistics of extracorporeal life support in 2018. Zhonghua Yi Xue Za Zhi. 2019;99(24):1911–5. 10.3760/cma.j.issn.0376-2491.2019.24.014. 31269589 10.3760/cma.j.issn.0376-2491.2019.24.014

[12] Vincent JL, Marshall JC, Namendys-Silva SA, et al. Assessment of the worldwide burden of critical illness: the intensive care over nations (ICON) audit. Lancet Respir Med. 2014;2(5):380–6. 10.1016/S2213-2600(14)70061-X. 24740011 10.1016/S2213-2600(14)70061-X

[13] Kashyap R, Hache-Marliere M, Gavrilovic S, Gajic O. Improving outcomes for the critically ill in developing countries: what is next? Rev Bras Ter Intensiva. 2015;27(4):312–4. 10.5935/0103-507X.20150054. 26761467 10.5935/0103-507X.20150054 PMC4738815

[14] Eyber R, Vaillancourt S, Perry W, Mannava P, Folaranmi T, Celi LA. Big data in global health: improving health in low- and middle-income countries. Bull World Health Organ 2015;93(3):203–8. 10.2471/BLT.14.139022. 25767300 10.2471/BLT.14.139022 PMC4339829

[15] Fraser HS, Blaya J. Implementing medical information systems in developing countries, what works and what doesn't. *AMIA Annu Symp Proc*. 2010;2010:232–236. Published 2010 Nov 13. PMC3041413 21346975

[16] Gupta A, Chawla A, Shushrutha KS, Mohana. Intelligent information retrieval: techniques for character recognition and structured data extraction. Int J Emerg Technol Innov Res. 2022;9(7):452–9.

[17] Yang X, Chen A, PourNejatian N, et al. A large language model for electronic health records. NPJ Digit Med. 2022;5(1):194. 10.1038/s41746-022-00742-2. 36572766 10.1038/s41746-022-00742-2 PMC9792464

[18] Eradat Oskoui S, Retford M, Forde E, et al. Developing a prototype for federated analysis to enhance privacy and enable trustworthy access to COVID-19 research data. Int J Med Inform. 2025;195: 105708. 10.1016/j.ijmedinf.2024.105708. 39642590 10.1016/j.ijmedinf.2024.105708

