# Supplementary material for: Streamlining data recording through optical character recognition: a prospective multi-center study in intensive care units
Source: Crit Care. 2025 Mar 18;29:117. doi: 10.1186/s13054-025-05347-1 (PMC11917072; doi:10.1186/s13054-025-05347-1)
Supplement: Supplementary file 1 — Additional file 1. [file 13054_2025_5347_MOESM1_ESM.docx]

**Streamlining Data Recording through Optical Character Recognition: A Prospective Multi-Center Study in Intensive Care Units**

**Supplemental Table of Contents**

**Supplemental Methods** Technical description of development of optical character recognition-based system. 2

**Supplemental Table 1.** Type of monitors and devices, and amount of training and testing data for optical character recognition software development. 5

**Supplemental Table 2.** Target parameters in each of the monitors and devices. 6

**Supplemental Table 3.** Details of the user survey. 8

**Supplemental Table 4.** Data completeness and accuracy stratified by monitoring device. 9

**Supplemental Table 5.** Results from user survey. 10

**Supplemental Figure 1.** Labeled photos of physiological monitors. 11

**Supplemental Figure 2.** Labeled photos of hemodynamic monitors. 13

**Supplemental Figure 3.** Labeled photos of mechanical ventilators. 14

**Supplemental Figure 4.** Labeled photos of extracorporeal membrane oxygenation. 18

**Supplemental Figure 5.** Labeled photos of laboratory results. 21

**Supplemental Methods. Technical description of development of optical character recognition-based system.**

The OCR system operates in four sequential stages: preprocessing (Image Localization), text detection, text recognition, and key information extraction. While end-to-end approaches using Document Understanding Transformer (Donut) [1] or Large Vision-Language Model [2] have gained prominence recently, these methods require extensive training data for handling specialized fonts and diverse formats, and present challenges in incorporating detailed meta-information about possible value ranges.


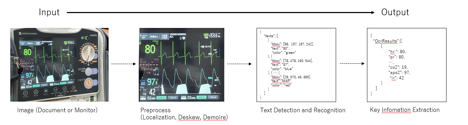


1. Preprocessing

Due to the presence of external monitors, power cords in the background or foreground, and non-frontal capturing conditions, we implemented a two-step preprocessing approach. First, we trained Yolact [3], an Instance Segmentation Model, on our proprietary dataset to localize the target device and perform dewarping correction. Additionally, since our OCR targets are susceptible to moiré patterns, we applied demoiré processing to images with significant noise.


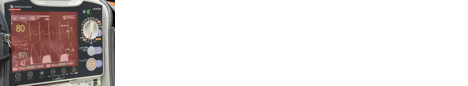


2. Text Detection and Recognition

The majority of text in the images is processed using a two-stage approach: text string detection with CRAFT [4] followed by recognition using CRNN [5]. Due to the presence of specialized fonts in our target devices, we developed a custom program to generate similar cases for training the CRNN recognition component. While this approach is effective for most characters, we encountered two specific challenges: (1) small but crucial phrases going undetected, and (2) inherently illegible characters, such as the "e" in "VTe" shown in below Figure. To address these limitations, we developed a supplementary model focusing on detecting only critical keywords, which we merged with the CRAFT+CRNN results. We utilized RTMDet [6], an object detection model particularly effective for small objects, training it to directly detect specific terms (e.g., "VTi", "VTe", "PS above PEEP", "PC above PEEP", "f") from monitor images, thereby complementing the primary CRAFT+CRNN system.


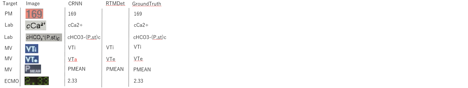


3. Key Information Extraction (KIE)

The final stage involves extracting target keywords and their corresponding values using a rule-based system that considers multiple parameters: character size, layout, surrounding text, positional relationships within the screen, value magnitudes, and in PM cases, character colors. We maintain multiple layout and notation rules for each device model, with the most comprehensive extraction result being selected as the final OCR output.

References

1. Kim, Geewook, et al. "Ocr-free document understanding transformer." European Conference on Computer Vision. Cham: Springer Nature Switzerland, 2022.
2. Wei, Haoran, et al. "General ocr theory: Towards ocr-2.0 via a unified end-to-end model." (2024).
3. Bolya, Daniel, et al. "Yolact: Real-time instance segmentation." Proceedings of the IEEE/CVF international conference on computer vision. 2019.
4. Baek, Youngmin, et al. "Character region awareness for text detection." Proceedings of the IEEE/CVF conference on computer vision and pattern recognition. 2019.
5. Shi, Baoguang, Xiang Bai, and Cong Yao. "An end-to-end trainable neural network for image-based sequence recognition and its application to scene text recognition." IEEE transactions on pattern analysis and machine intelligence 39.11 (2016): 2298-2304.
6. Lyu, Chengqi, et al. "Rtmdet: An empirical study of designing real-time object detectors." arXiv preprint arXiv:2212.07784(2022).

**Supplemental Table 1. Type of monitors and devices, and amount of training and testing data for OCR software development.**

| Monitor type | Model name | Country | Number of photos for OCR development | Number of photos for OCR validation | Number of photos for OCR test |
| --- | --- | --- | --- | --- | --- |
| Physiological monitor* | GE HealthCare CARESCAPE Bx50 monitor SW v3 | AU | 50 | 31 | 9 |
|  | PHILIPS IntelliVue MX800 | HK | 81 | 26 | 8 |
|  | PHILIPS IntelliVue MP70 | TH | 38 | 26 | 5 |
|  | PHILIPS IntelliVue MX550 | TH | 18 | 26 | 3 |
| Hemodynamic monitor | GE HealthCare CARESCAPE Bx50 monitor SW v3 | AU | 50 | 26 | 8 |
|  | Edwards Lifesciences VigilanceⅡ | AU | 59 | 26 | 0 |
| Mechanical ventilator | HAMILTON MEDICAL G-5 | AU | 154 | 29 | 8 |
|  | Maquet Servo-u | HK | 35 | 59 | 8 |
|  | Maquet Servo-i | HK | 17 | 12 | 0 |
|  | Drager Evita XL | HK | 34 | 35 | 4 |
|  | Nellcor Puritan Bennett 840 | TH | 31 | 35 | 4 |
| ECMO | MERA HCS-CFP | AU | 30 | 31 | 8 |
|  | MAQUET Cardiohelp | HK | 60 | 30 | 8 |
|  | MAQUET Rotaflow | HK | 53 | 0 | 1 |
|  | Xenios | TH | 48 | 0 | 7 |
| Laboratory data | RADIO METER ABL 90SERIES | AU | 30 | 29 | 8 |
|  | Philips IntelliSpace Console Critical Care | HK | 50 | 24 | 8 |
|  | Unknown | TH | 30 | 24 | 9 |

Abbreviations: AU, Australia; ECMO, extracorporeal membrane oxygenation; HK, Hong Kong; TH, Thailand.

* Some types of physiological monitors include the option to display hemodynamic parameters.

**Supplemental Table 2. Target parameters in each of the monitors and devices.**

| **Physiological or hemodynamic monitors** | **Mechanical ventilator** | **Extracorporeal membrane oxygenation** | **Laboratory data** |
| --- | --- | --- | --- |
| Systolic blood pressure | Mode of ventilation | Pump speed | Hemoglobin |
| Diastolic blood pressure | FiO2 | Blood flow rate | Hematocrit |
| Mean arterial pressure | RR set | pArt | WBC |
| Heart rate | Peak inspiratory pressure | plnt | Platelet |
| PA systolic pressure | PEEP | pVen | PT |
| PA diastolic pressure | Tidal volume | Delta P | APTT |
| PA mean pressure | Pressure control | Temperature | INR |
| Cardiac output | Pressure support | SvO2 | Fibrinogen |
| Cardiac index | RR delivered |  | D-dimer |
| PCWP | Ppeak |  | Sodium |
| Central venous pressure | Pplat |  | Potassium |
| Respiratory rate | Pmean |  | Chloride |
| SpO2 | PEEP delivered |  | Blood urea nitrogen |
| SvO2 | TV inspired |  | Creatinine |
| etCO2 | TV expired |  | Bilirubin |
| Body temperature | Minute ventilation |  | Total protein |
| Systemic vascular resistance |  |  | Albumin |
| Pulmonary vascular resistance |  |  | ALP |
|  |  |  | ALT |
|  |  |  | AST |
|  |  |  | Calcium |
|  |  |  | Phosphate |
|  |  |  | Glucose |
|  |  |  | Lactate |
|  |  |  | pH |
|  |  |  | PaO2 |
|  |  |  | PaCO2 |
|  |  |  | Bicarbonate |
|  |  |  | Base excess |

Abbreviations: ALP, alkaline phosphatase; ALT, alanine transferase; APTT, activated partial thromboplastin time; AST, aspartate transferase; Delta P, difference between pArt and pInt; etCO2, end-tidal carbon dioxide; FiO2, fractional inspired oxygen; INR, international normalized ratio; PA, pulmonary artery; PaCO2, partial pressure of carbon dioxide; PaO2, partial pressure of oxygen; pArt, pressure in arterial limb; PCWP, pulmonary capillary wedge pressure; PEEP, positive end-expiratory pressure; Pmean, mean inspiratory pressure; pInt, internal pressure; Ppeak, peak inspiratory pressure; Pplat, plauteu inspiratory pressure; PT, prothrombin time; pVen, pressure in venous limb; RR, respiratory rate; SpO2, oxygen saturation; SvO2, venous oxygen saturation; TV, tidal volume; WBC, white blood cell.

**Supplemental Table 3. Details of the user survey.**

|  | **Question** | **Options** |
| --- | --- | --- |
| 1 | Institution you belong to | 1. APELSO  2. Critical Care Research Group 3. School of Clinical Medicine, The University of Hong Kong  4. Siriraji Mahidol University  5. Other |
| 2 | How long did it take you to get used to using the EDC? | 1. I got used to it right away. 2. Got used to it within 1~2 weeks 3. Got used to it within a month 4. Still not used to it |
| 3 | How useful is the OCR function?　(*Function to output figures and characters accurately) | 1. Not useful - 5. Very useful |
| 4 | Are there any specific problems with the OCR function? | 1. No particular problem 2. Works slow 3. Stops working in the middle of the reading 4. Gets frozen 5. Low accuracy 6. Other |
| 5 | For which device interface does OCR function not work well?  (multiple choice) | Physiological monitor Hemodynamics monitor Mechanical ventilator Extracorporeal membrane oxygenation Laboratory Results |
| 6 | Will the OCR function save time to enter patient data compared to manual entry? | 1. Not satisfied - 5. Very satisfied |
| 7 | Please tell us the reason. | Free text |
| 8 | What is your overall level of satisfaction of the EDC? | 1. Not satisfied - 5. Very satisfied |
| 9 | Would you recommend EDC to other research institutions? | 1. Not satisfied - 5. Very satisfied |
| 10 | Please let us know if you have any other suggestions or requests for improving the functions of the EDC. | Free text |

Abbreviations: APELSO, Asia-Pacific Extracorporeal Life Support Organization; EDC, electronic data capture (also known as case record form); OCR, optical character recognition.

**Supplemental Table 4. Data completeness and accuracy stratified by monitoring device.**

| Device Category | Monitoring Device | Number of Images | Number of Data Points | Data Completeness (%) | Data Accuracy (%) |
| --- | --- | --- | --- | --- | --- |
| Physiological Monitors | CARESCAPE Bx50 | 9 | 65 | 96.9 | 96.9 |
|  | IntelliVue MX800 | 12 | 63 | 95.2 | 82.5 |
|  | IntelliVue MP70 | 4 | 23 | 100.0 | 100.0 |
| Hemodynamic Monitors | CARESCAPE Bx50 | 10 | 80 | 97.5 | 95.0 |
| Mechanical Ventilators | Hamilton G5 | 8 | 71 | 100.0 | 100.0 |
|  | Maquet Servo-U | 8 | 96 | 99.0 | 97.9 |
|  | Evita XL | 4 | 45 | 100.0 | 100.0 |
|  | Bennet 840 | 4 | 44 | 100.0 | 100.0 |
| Extracorporeal Membrane Oxygenation (ECMO) Devices | HCS-CFP | 8 | 56 | 98.2 | 98.2 |
|  | Maquet CardioHelp | 8 | 64 | 100.0 | 95.3 |
|  | Maquet Rotaflow | 1 | 2 | 100.0 | 100.0 |
|  | Xenios | 7 | 49 | 100.0 | 100.0 |
| Blood Collection Data Monitoring | RADIOMETER ABL90 Series | 8 | 104 | 100.0 | 100.0 |
|  | Unnamed Device A | 8 | 117 | 98.3 | 97.4 |
|  | Unnamed Device B | 9 | 109 | 100.0 | 99.1 |

**Supplemental Table 5. Results from user survey.**

| **Responder** | **How long did it take you to get used to using the EDC?** | **How useful is the OCR function?** | **For which device interface does the OCR function not work well?** | **Will the OCR function save time to enter patient data compared to manual entry?** | **What is your overall level of satisfaction of the EDC?** | **Would you recommend EDC to other research institutions?** | **Are there any specific problems with the OCR function?** |
| --- | --- | --- | --- | --- | --- | --- | --- |
| 1 | I got used to it right away. | 5 |  | 5 | 4 | 5 | Works slow |
| 2 | I got used to it right away. | 3 | Mechanical ventilator, Laboratory Results | 4 | 3 | 2 | Low Accuracy, Works slow and unable to pickup most readings |
| 3 | Got used to it within 1~2 weeks | 5 | Laboratory Results | 5 | 5 | 5 | Stops working in the middle of the reading |
| 4 | I got used to it right away. | 4 | Physiological monitor | 5 | 4 | 5 | No particular problem |
| 5 | I got used to it right away. | 5 | Hemodynamics monitor | 5 | 5 | 5 | No particular problem |
| 6 | I got used to it right away. | 5 | ECMO | 5 | 3 | 4 | Low accuracy |
| 7 | I got used to it right away. | 5 | Physiological monitor, Hemodynamics monitor, Mechanical ventilator, Laboratory Results | 5 | 4 | 4 | Works slow |
| 8 | I got used to it right away. | 4 | Laboratory Results | 5 | 4 | 4 | Works slow |

Abbreviations: APELSO, Asia-Pacific Extracorporeal Life Support Organization; EDC, electronic data capture (also known as case record form); OCR, optical character recognition.

**Supplemental Figure 1. Labeled photos of physiological monitors.**

**
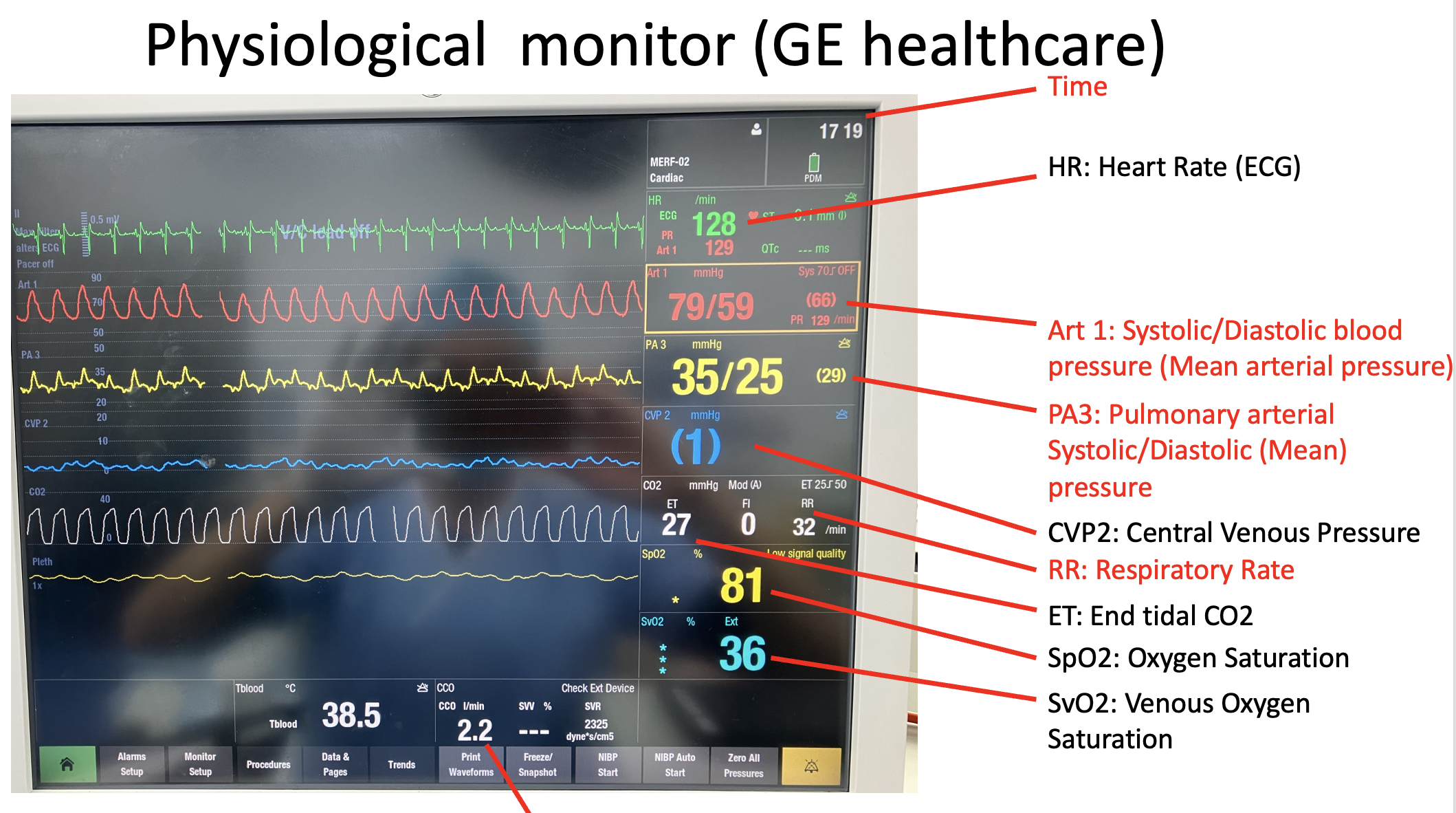
**

Panel A, GE Healthcare CARESCAPE Bx50 in Australia.


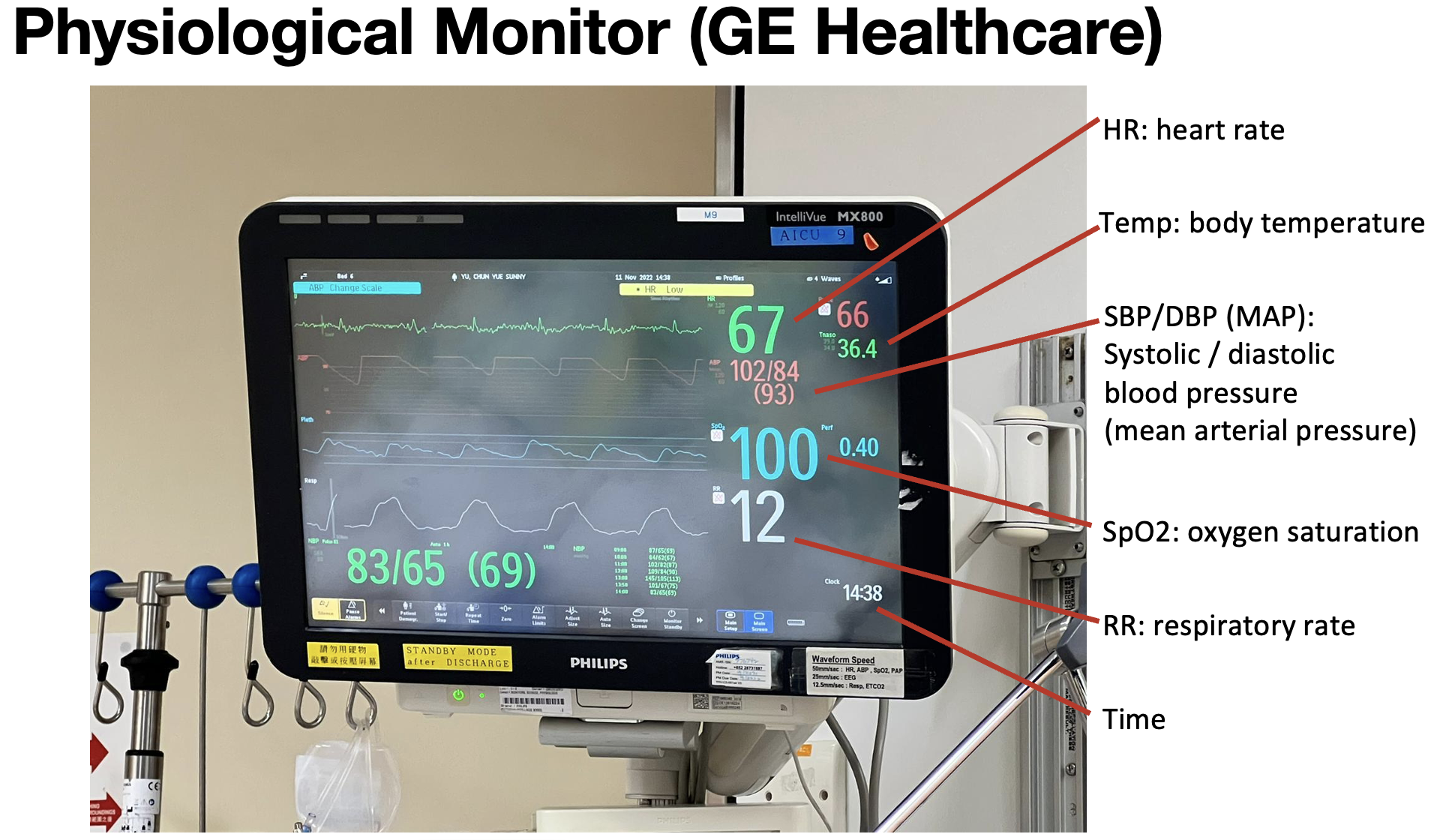


Panel B, Philips IntelliVue MX 800 in Hong Kong.


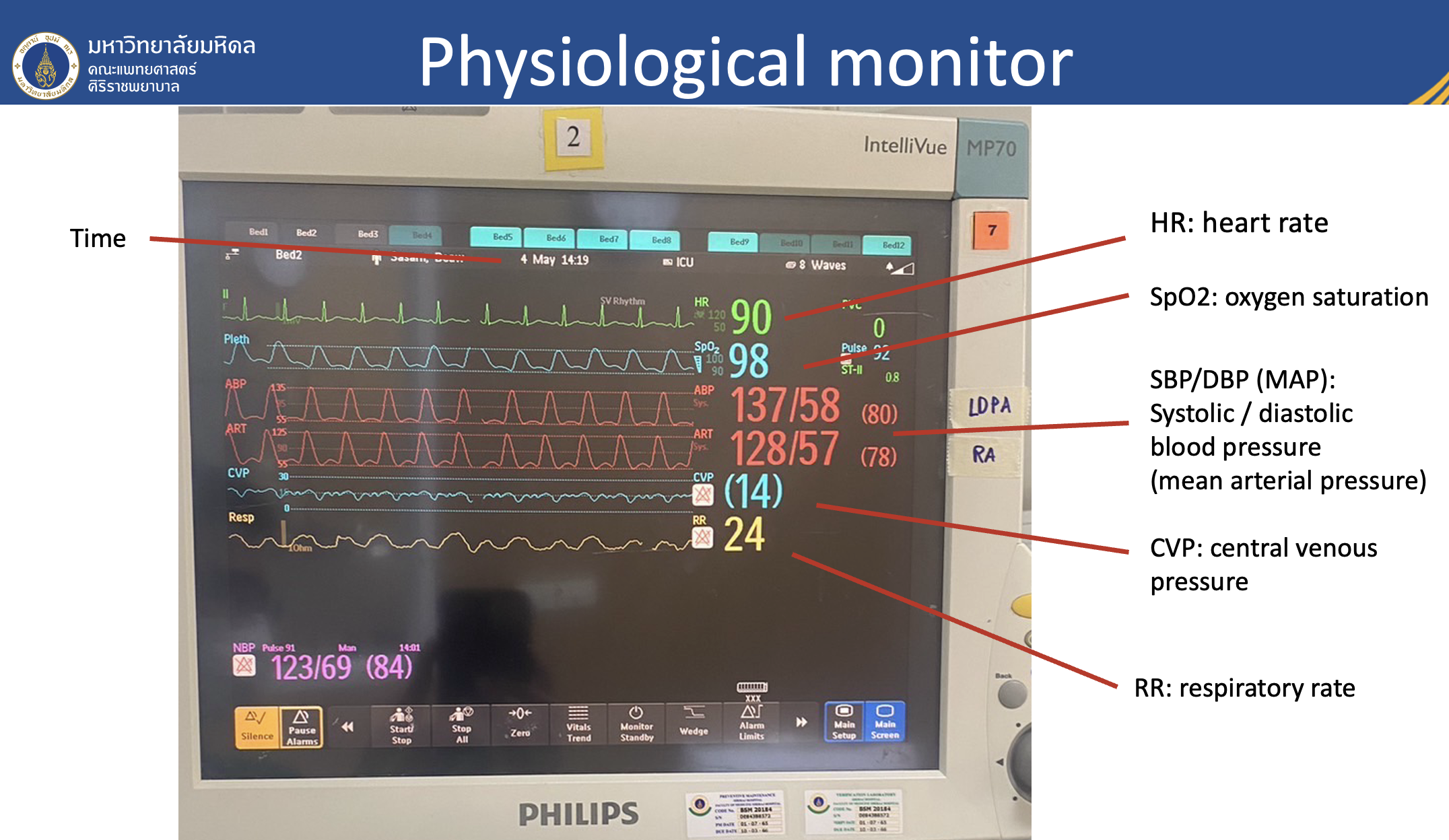


Panel C, Philips IntelliVue MP70 in Thailand.


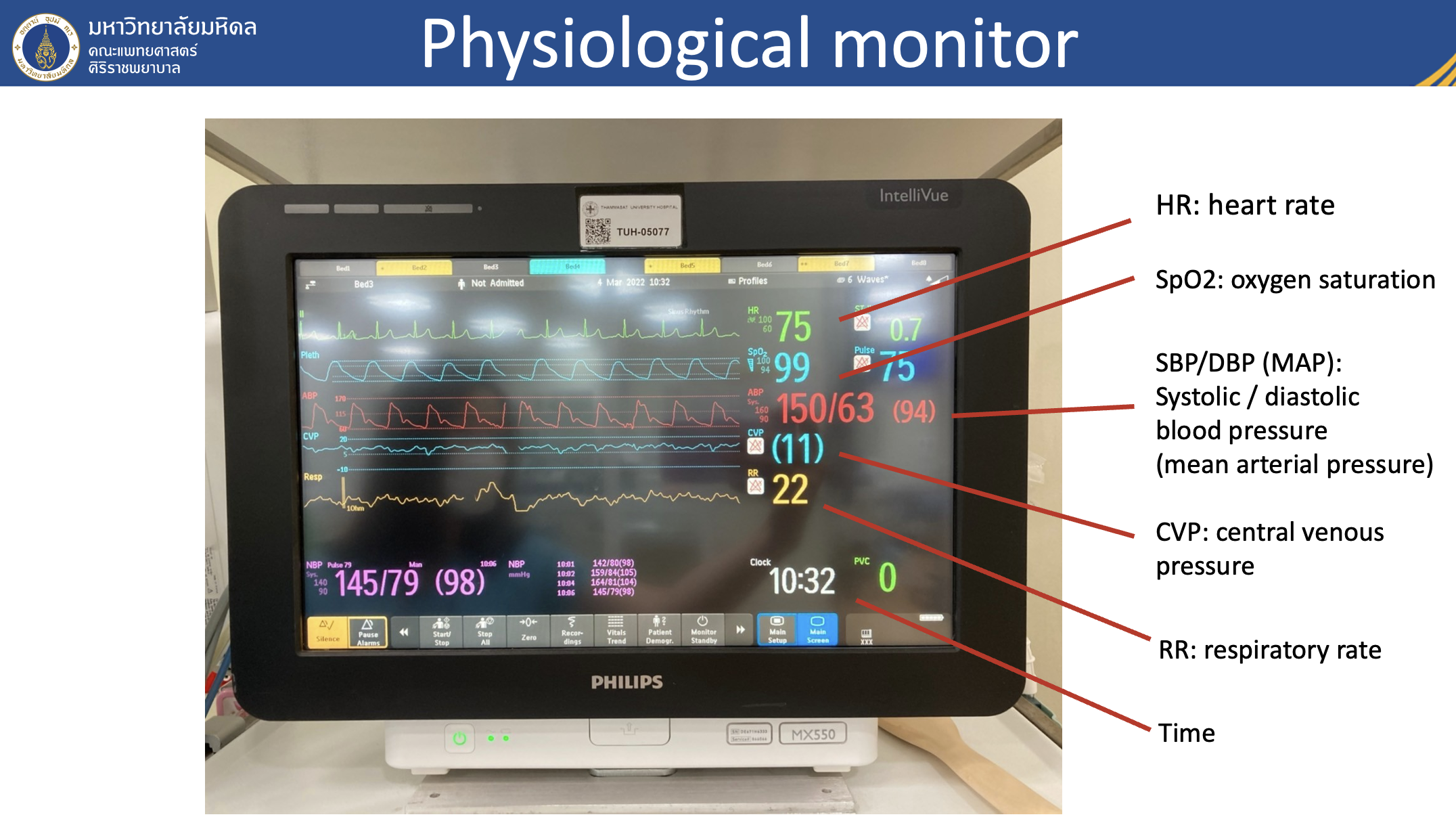


Panel D, Philips IntelliVue MX550 in Thailand.

**Supplemental Figure 2. Labeled photos of hemodynamic monitors.**

**
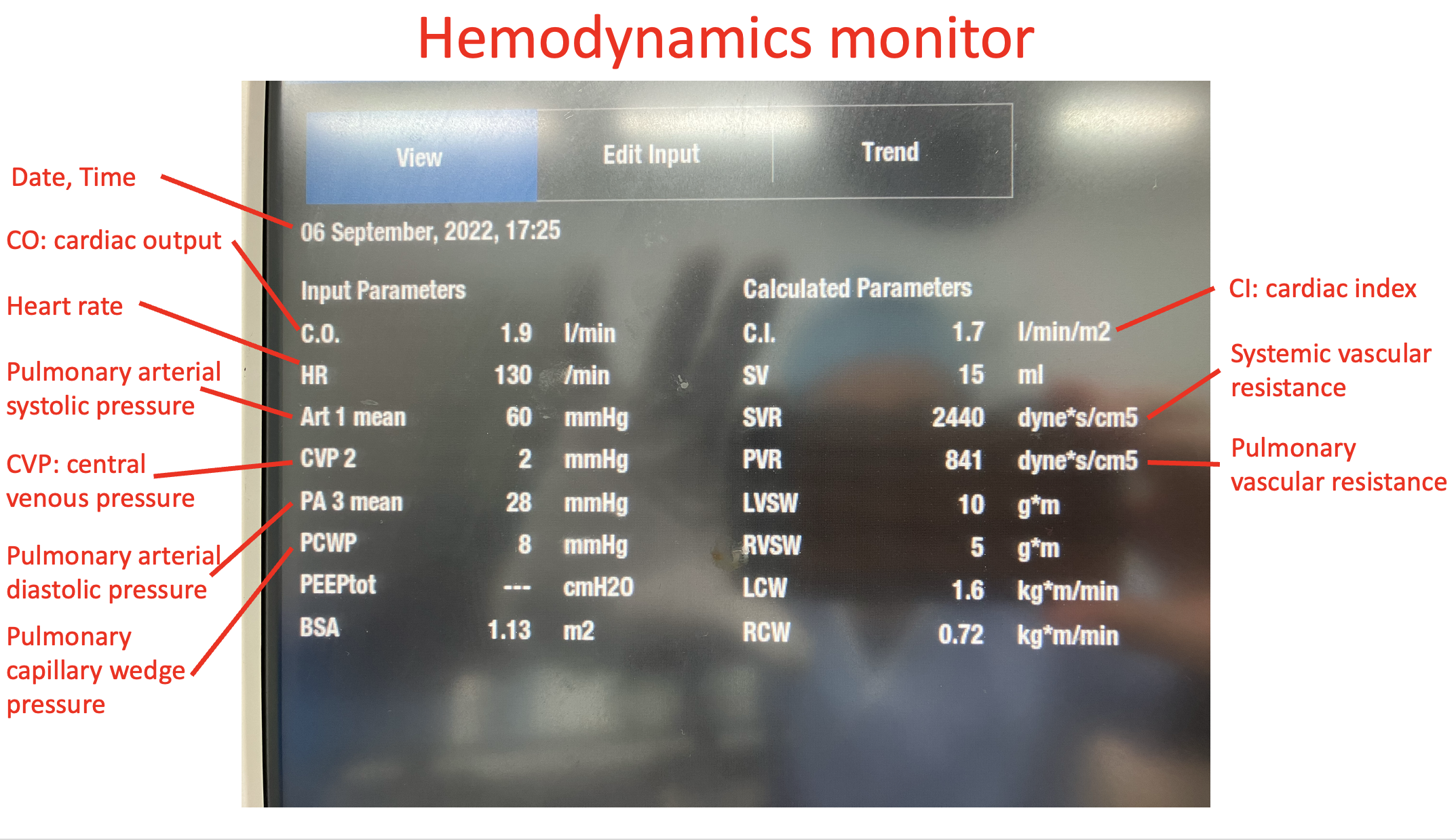
**

Panel A, GE Healthcare CARESCAPE Bx50 in Australia.

**
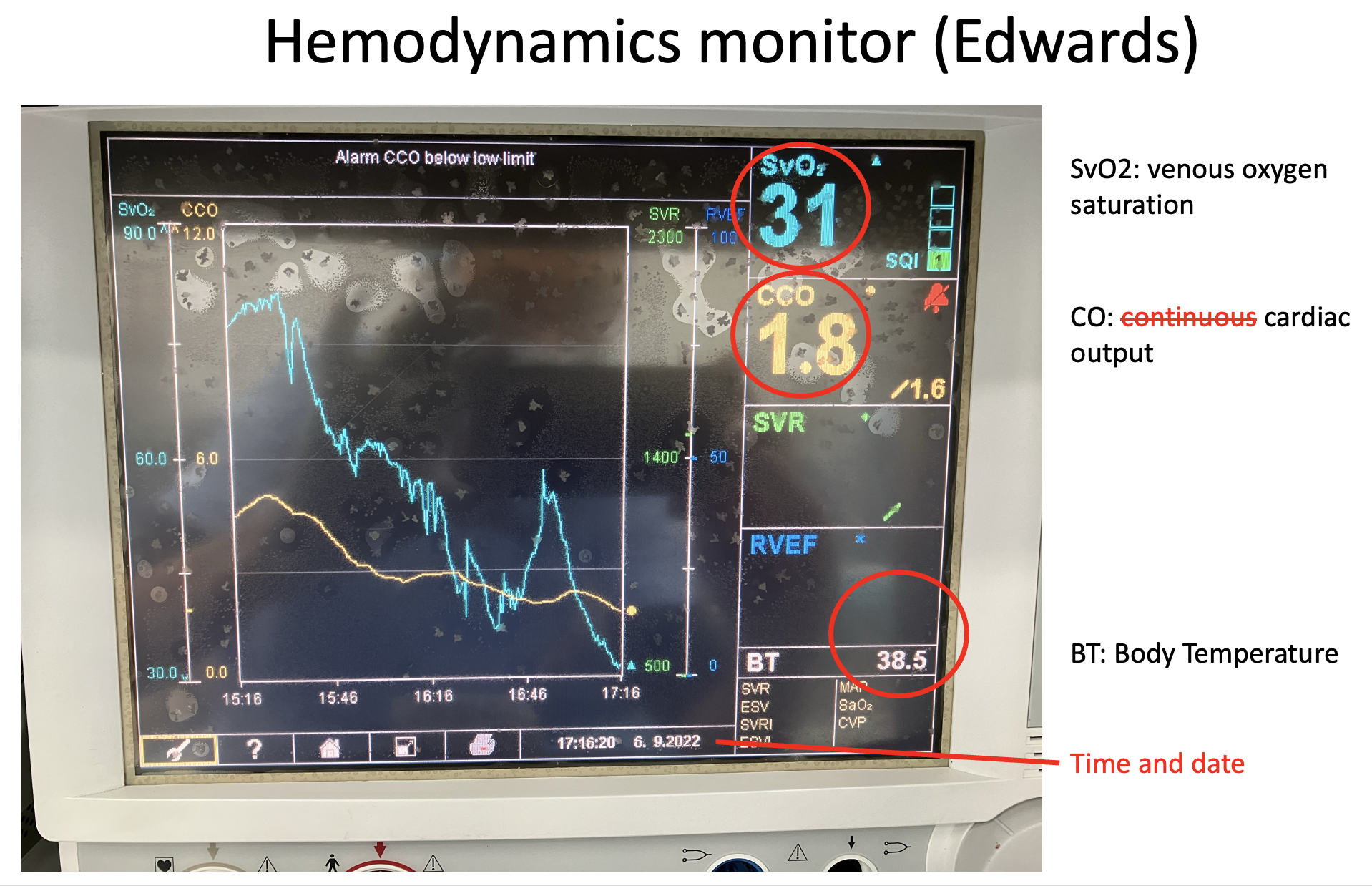
**

Panel B, Edwards Lifesciences VigilanceII in Australia.

**Supplemental Figure 3. Labeled photos of mechanical ventilators.**


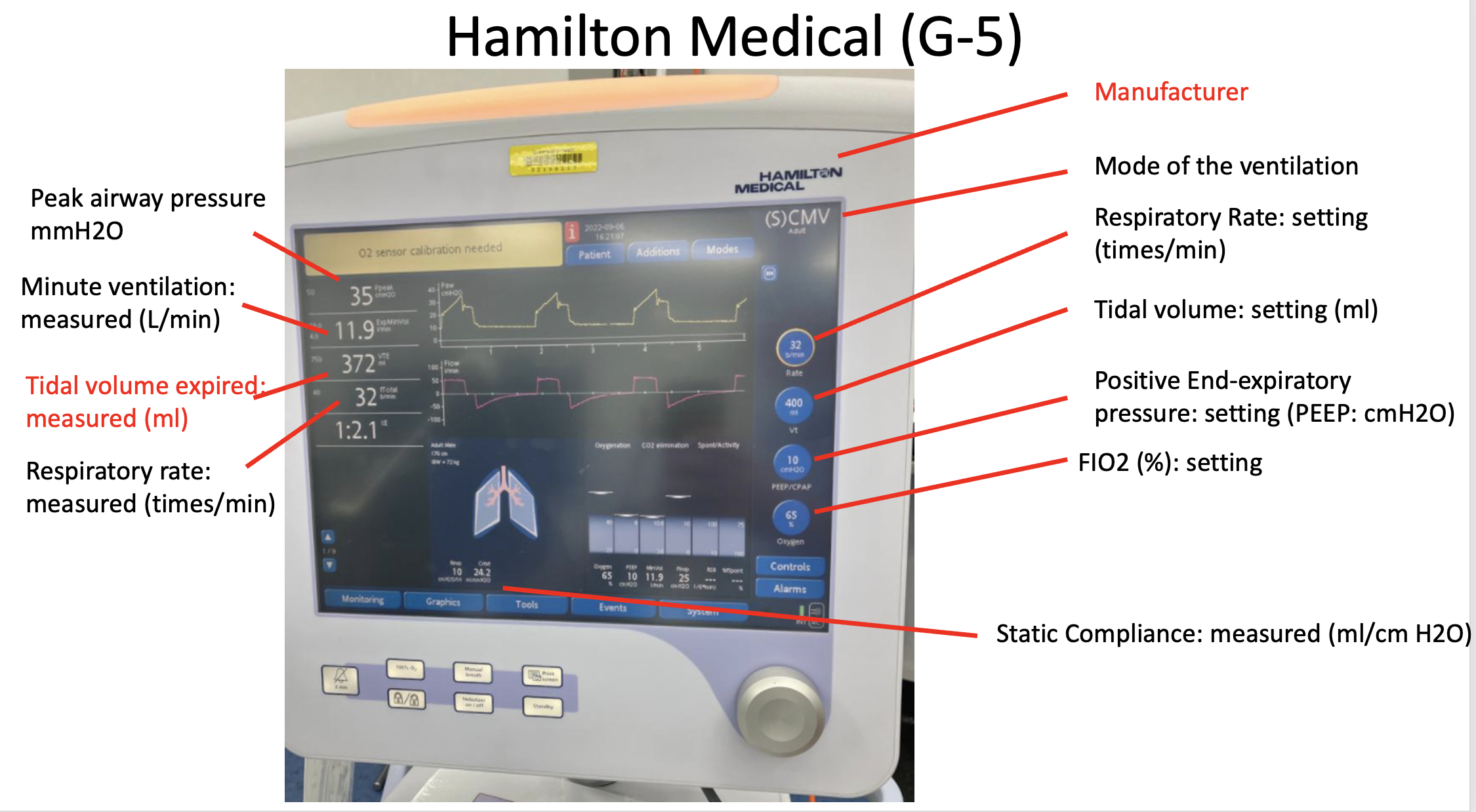


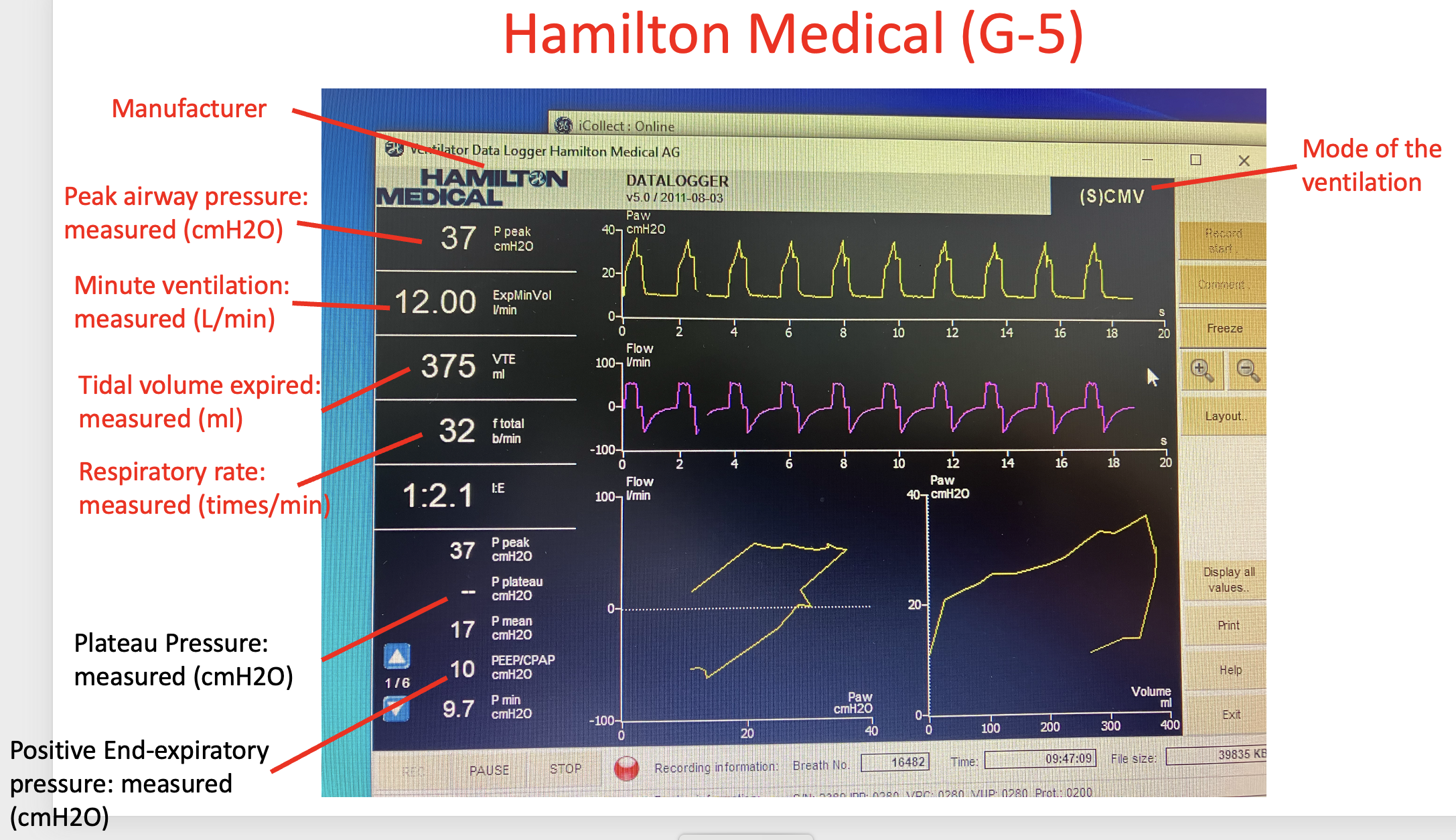


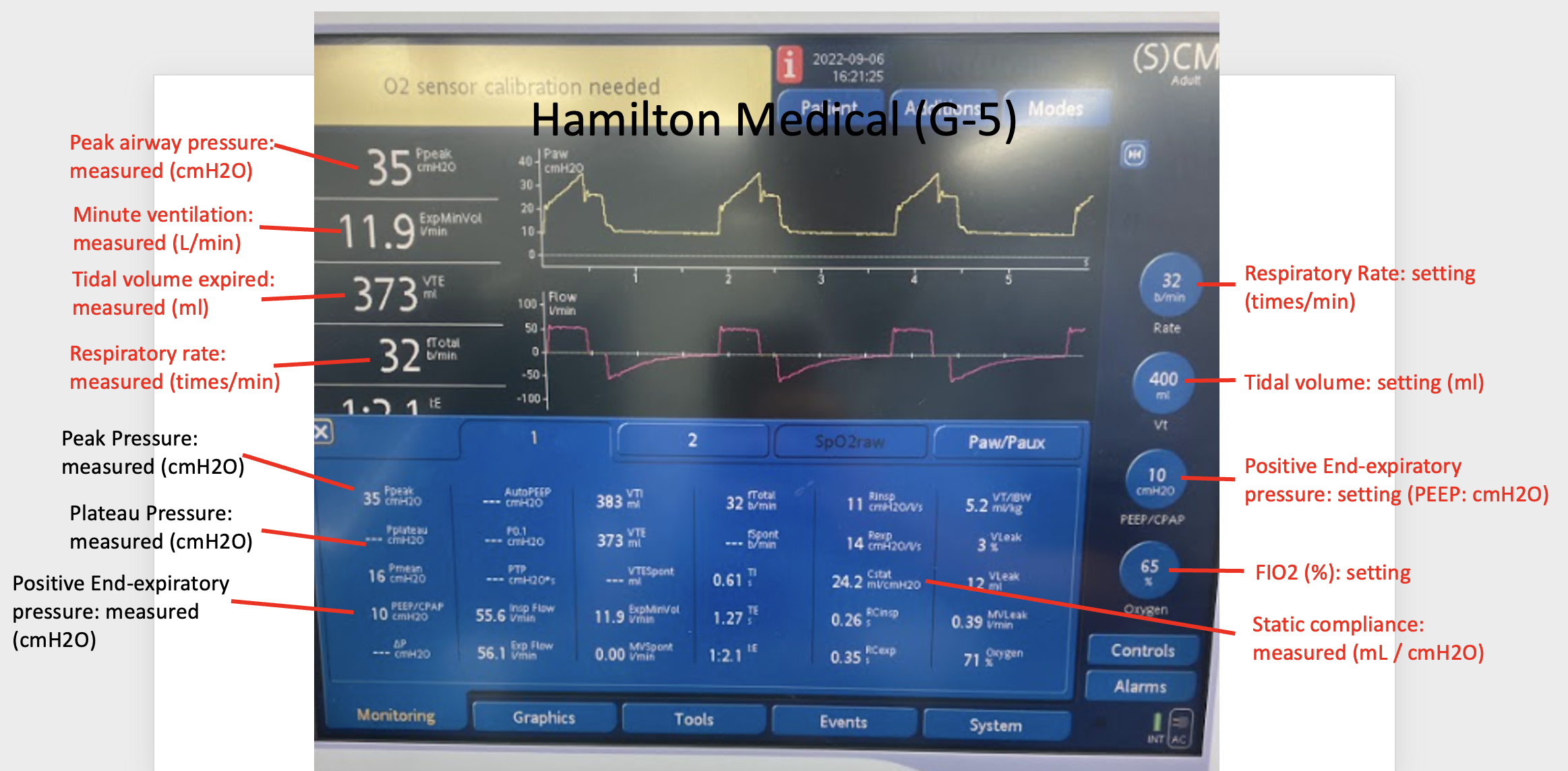


Panel A, Hamilton Medical G-5 in Australia.


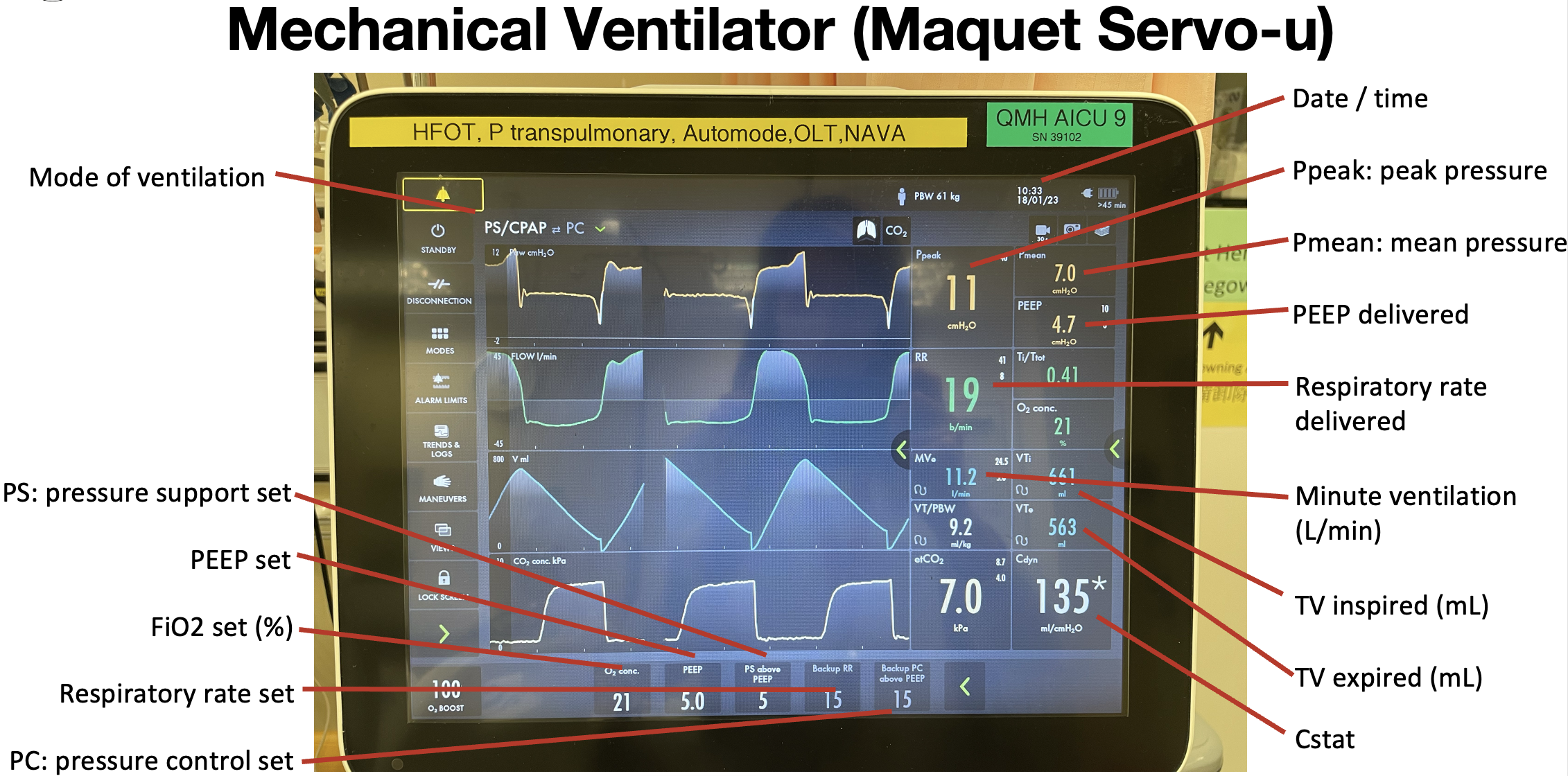


Panel B, Maquet Servo-u in Hong Kong.


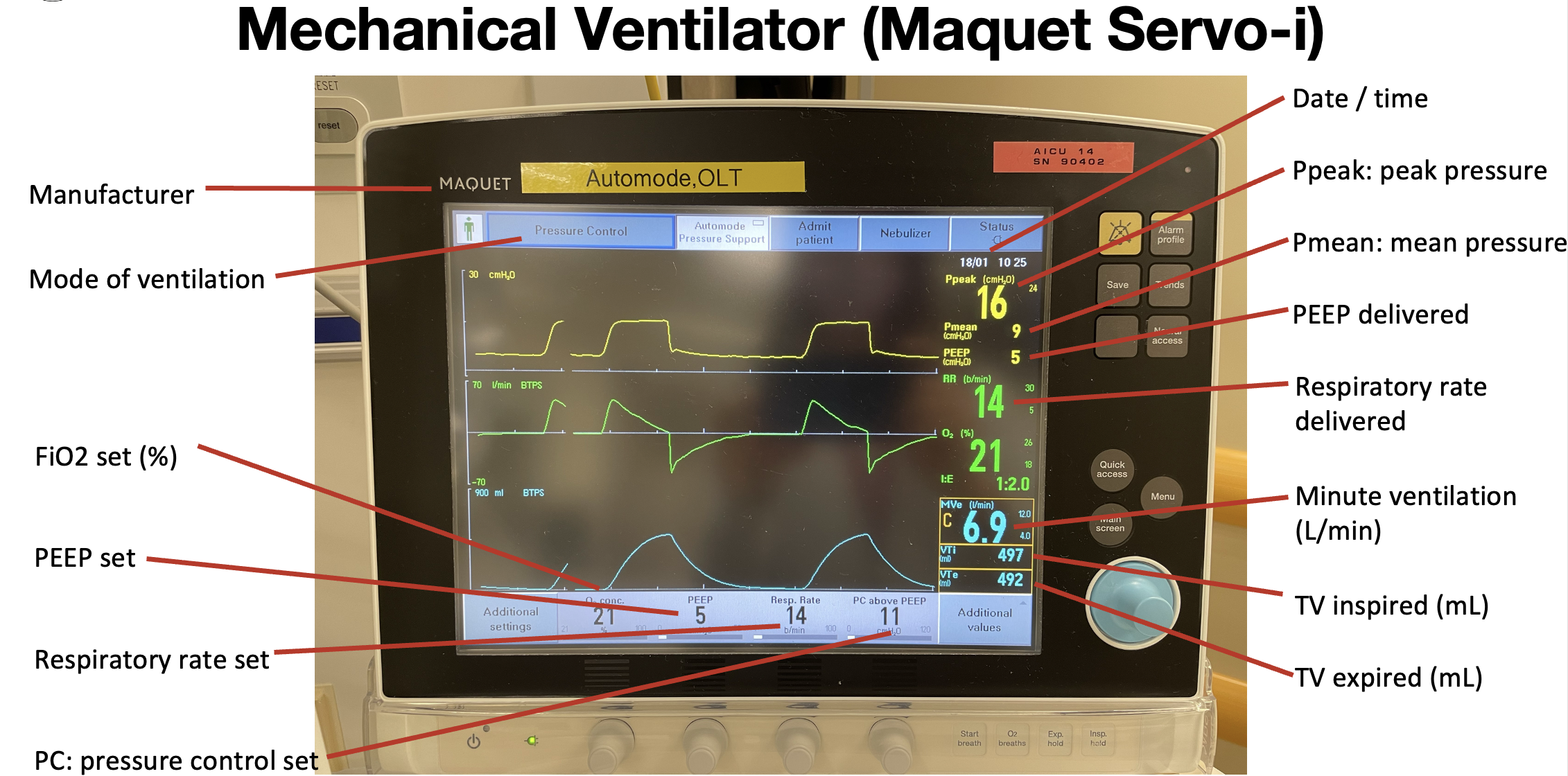


Panel C, Maquet Servo-i in Hong Kong.


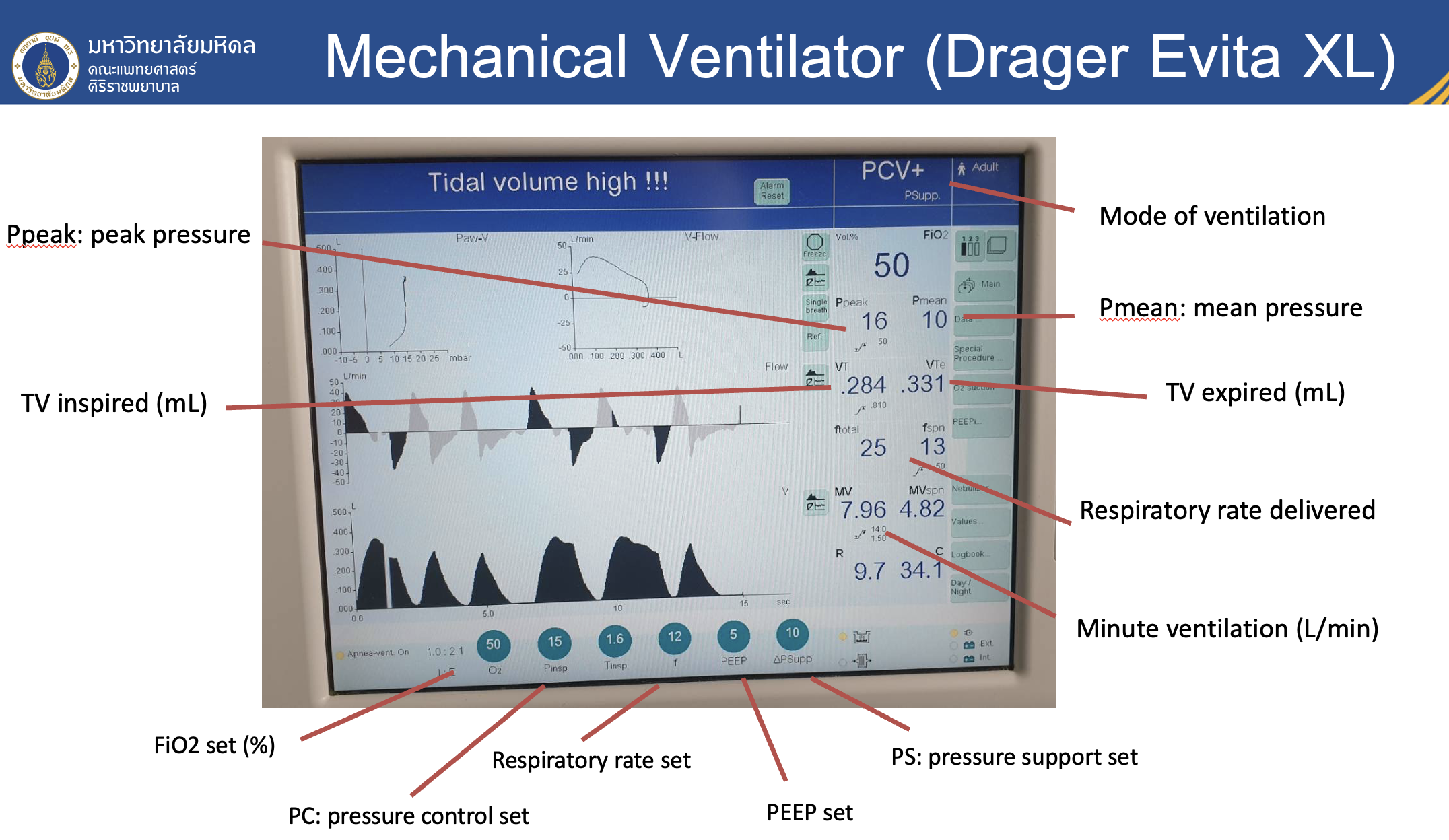


Panel D, Drager Evita XL in Thailand.


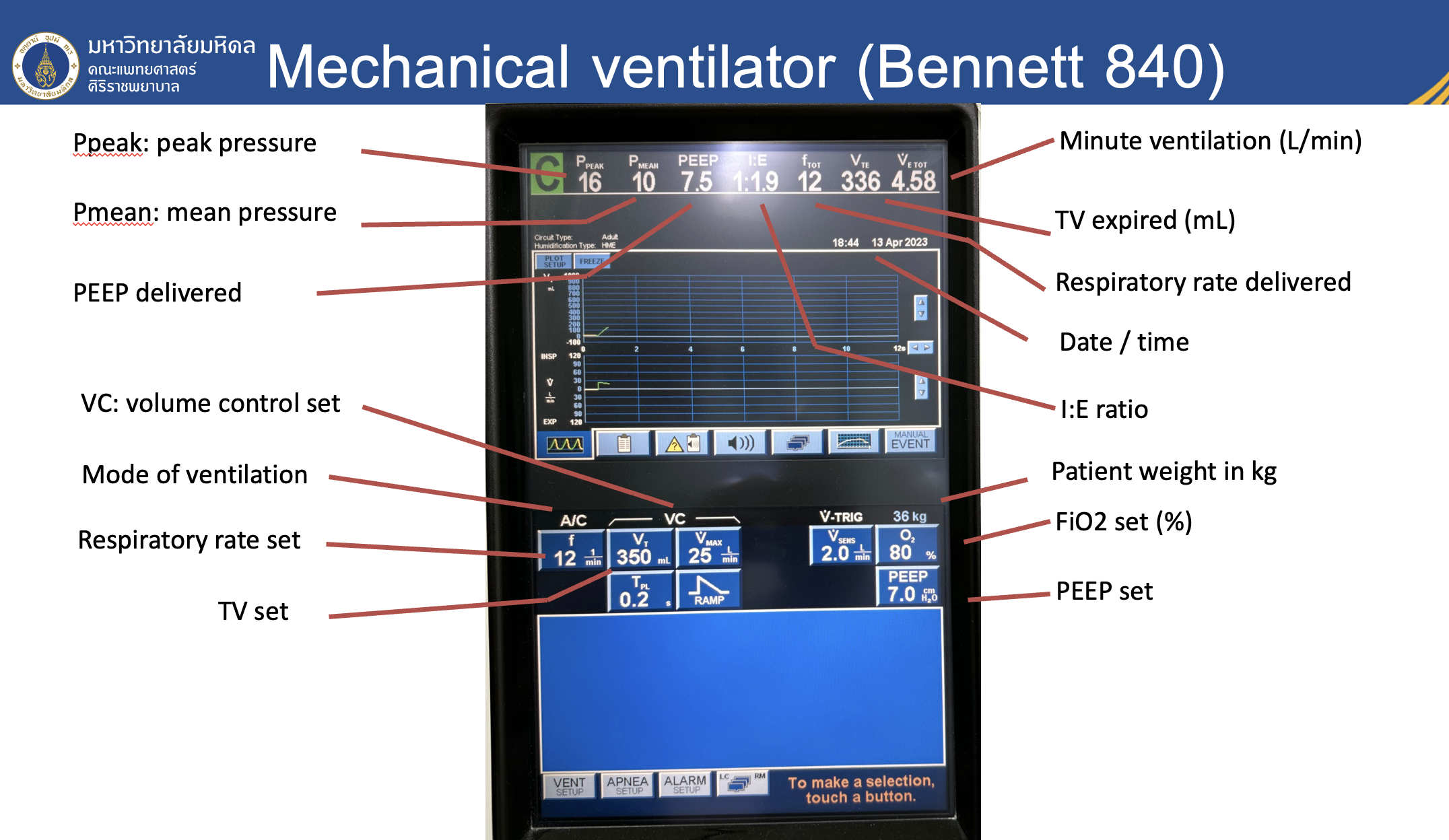


Panel E, Nellcor Puritan Bennett 840 in Thailand.

**Supplemental Figure 4. Labeled photos of extracorporeal membrane oxygenation.**


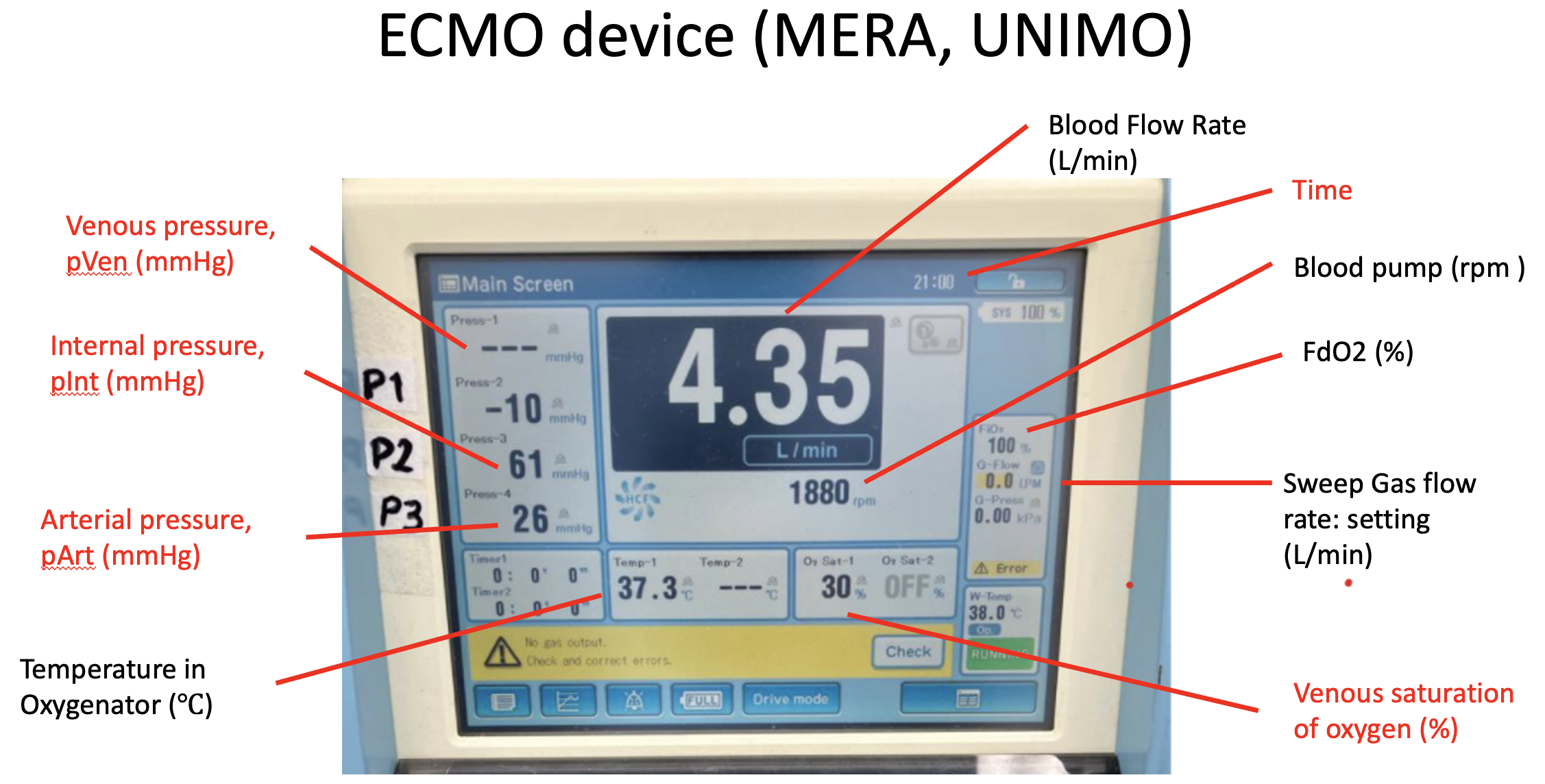


Panel A, Mero HCS-CFP in Australia.


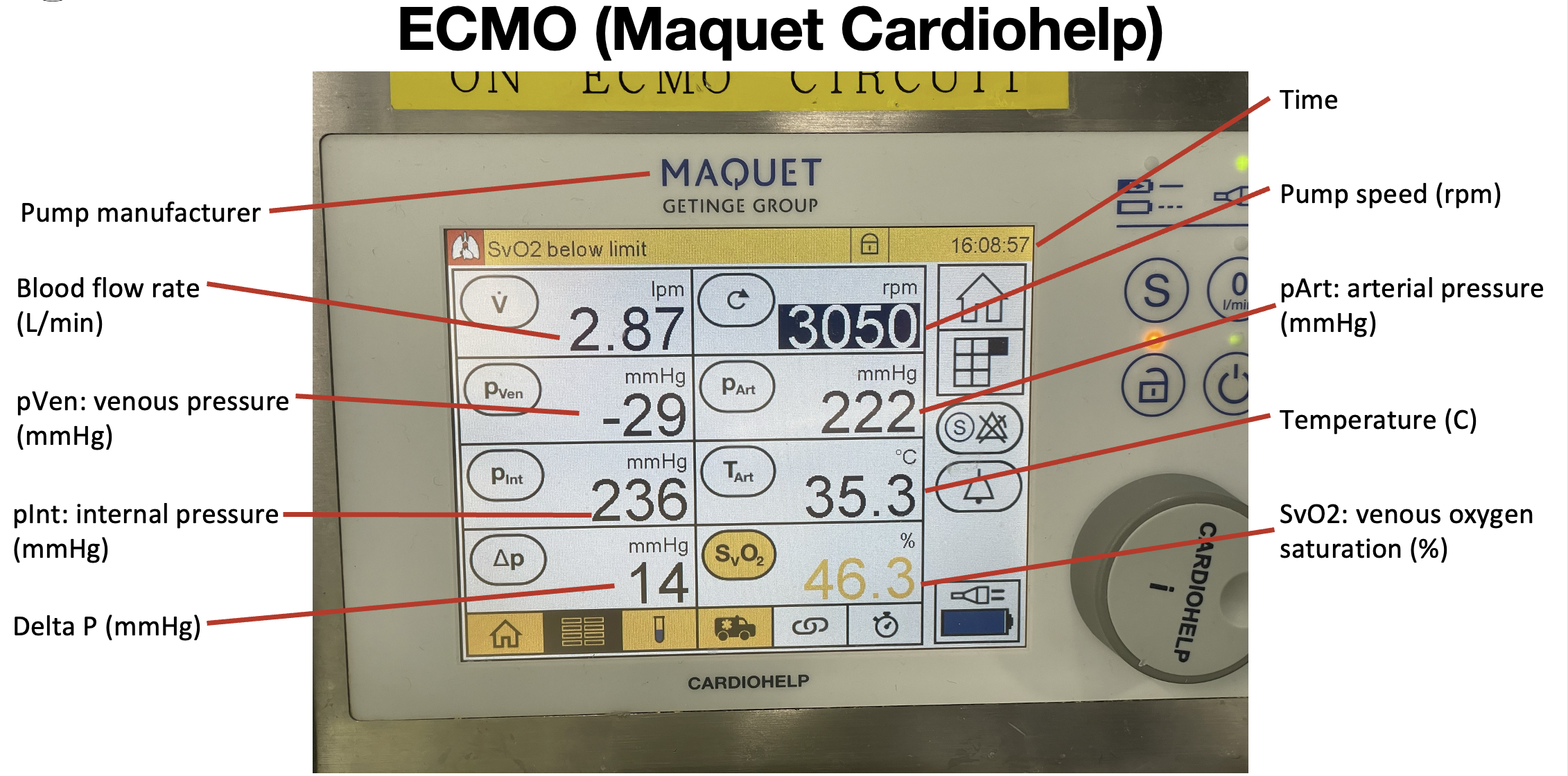


Panel B, Maquet Cardiohelp in Hong Kong.


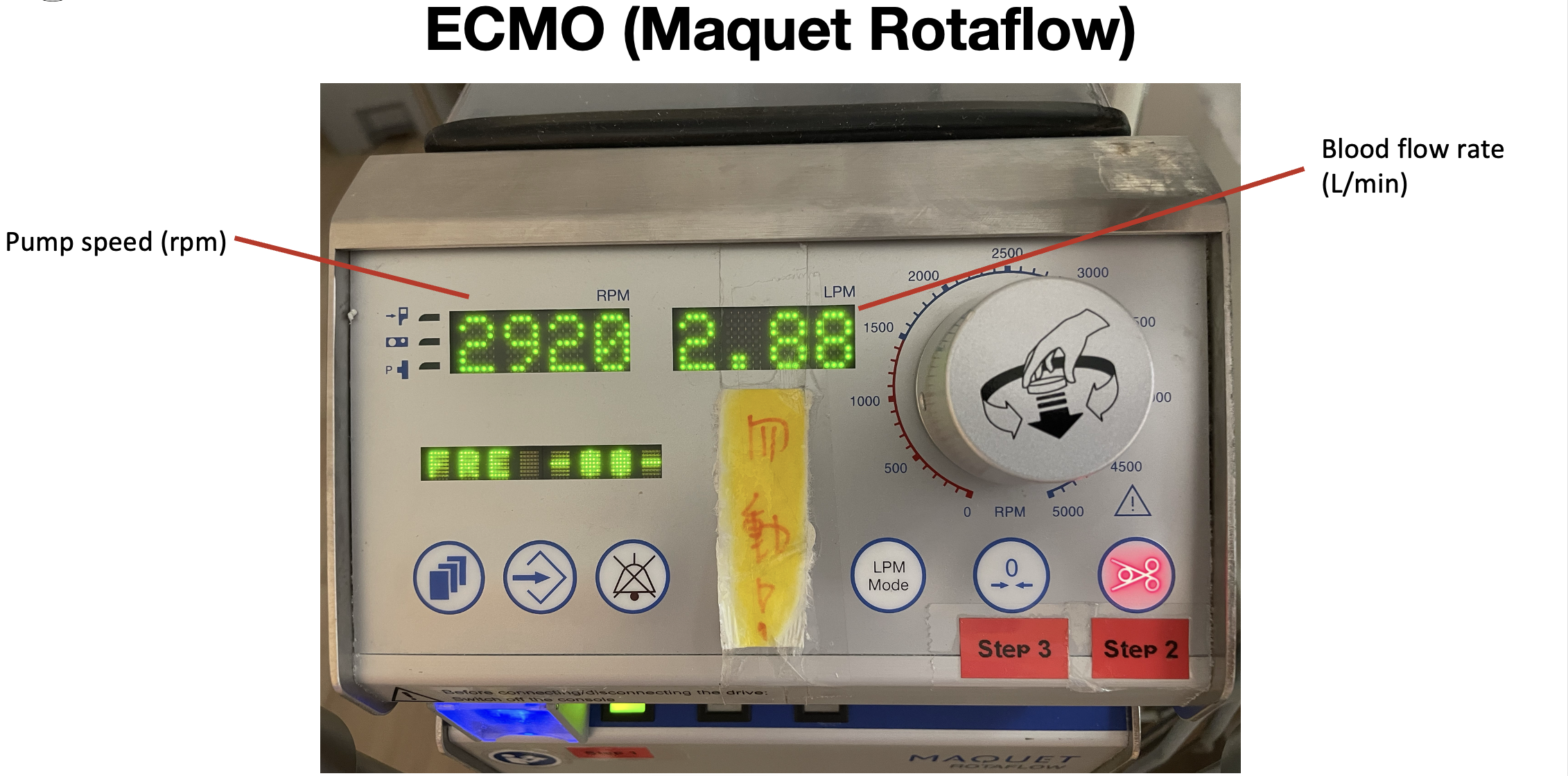


Panel C, Maquet Rotaflow in Hong Kong.


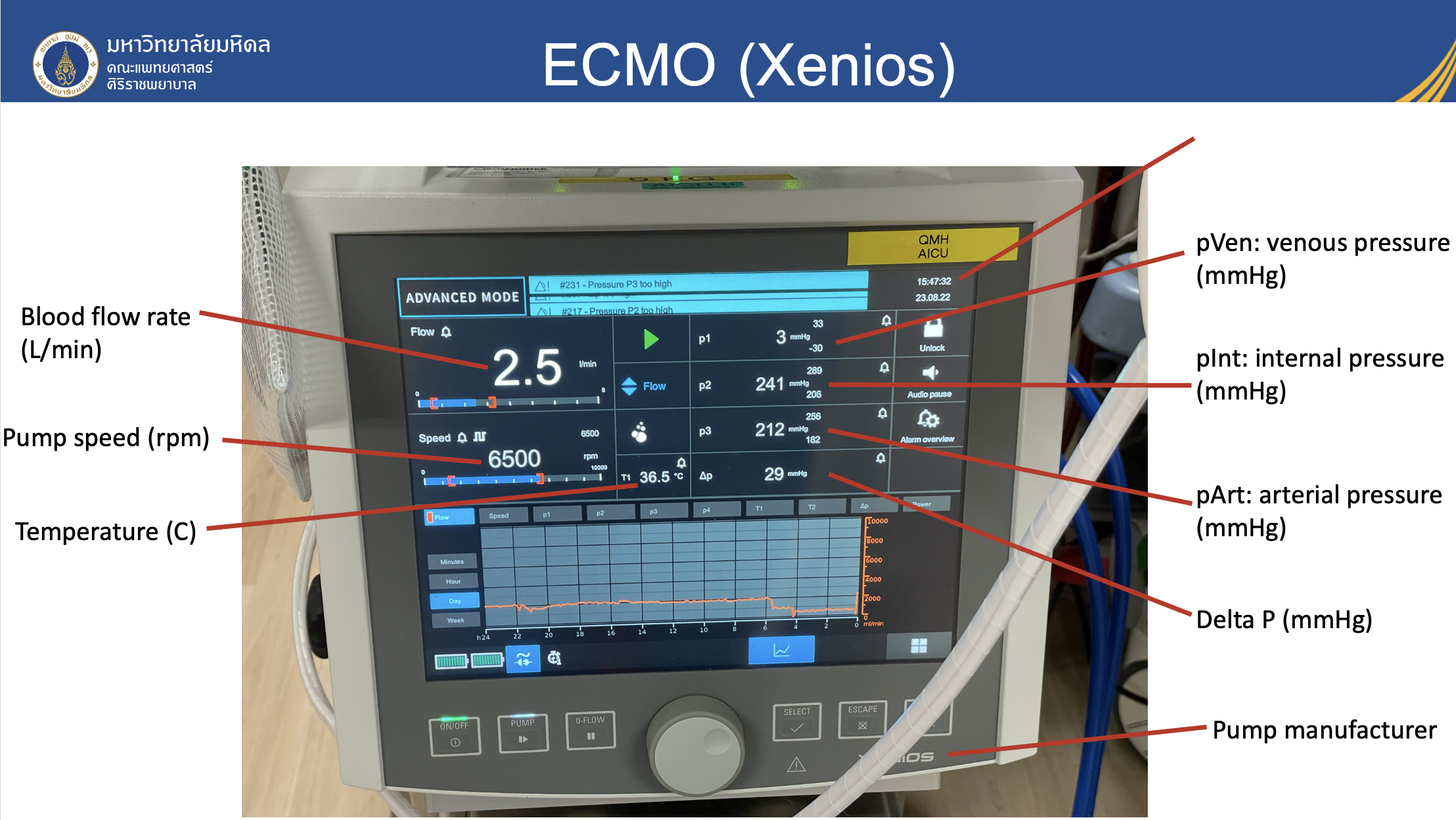


Panel D, Xenios in Thailand.


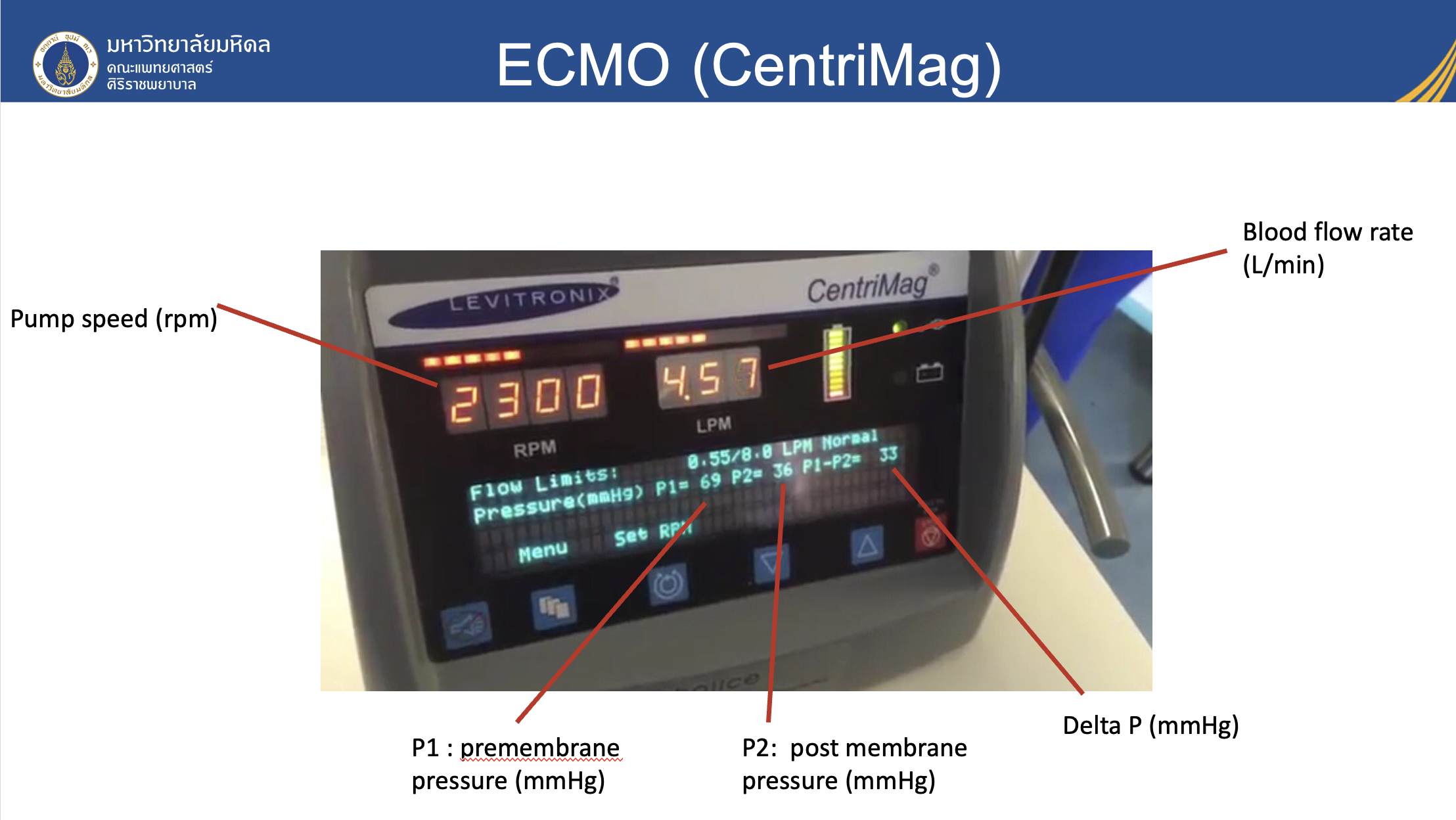


Panel E, CentriMag in Thailand.


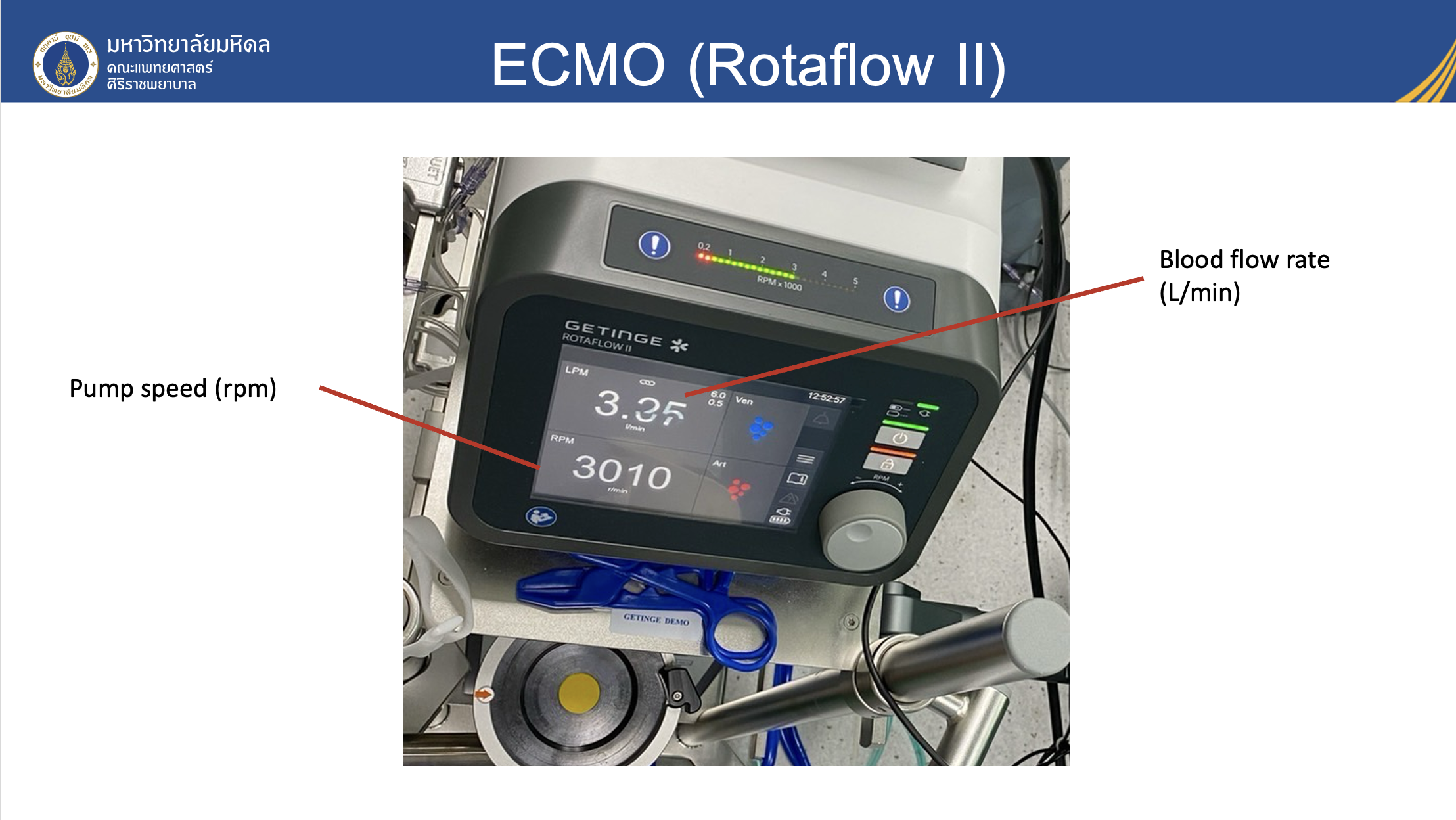


Panel F, Maquet Rotaflow II in Thailand.

**Supplemental Figure 5. Labeled photos of laboratory results.**


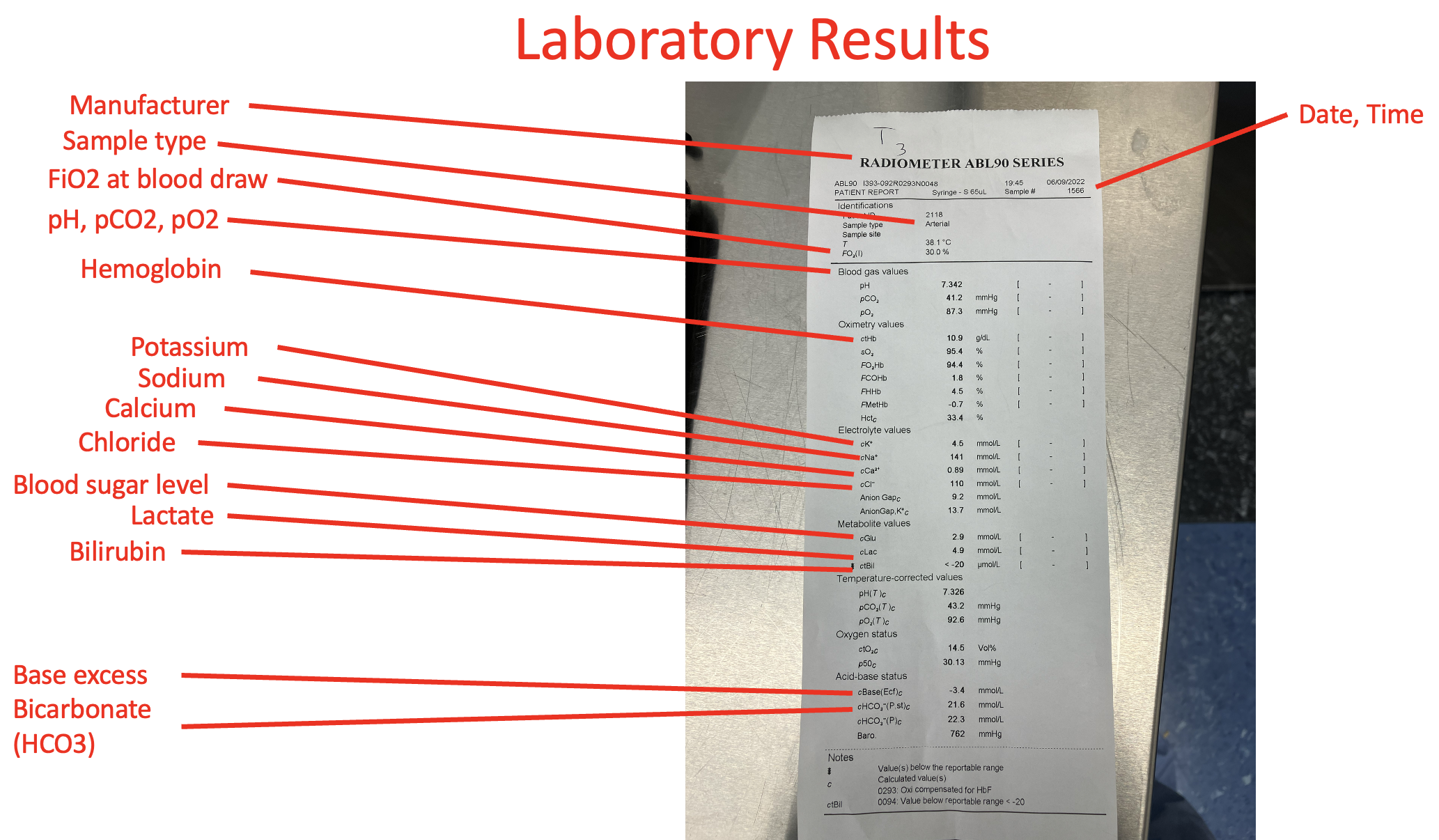


Panel A, RADIOMETER ABL 90SERIES in Australia.


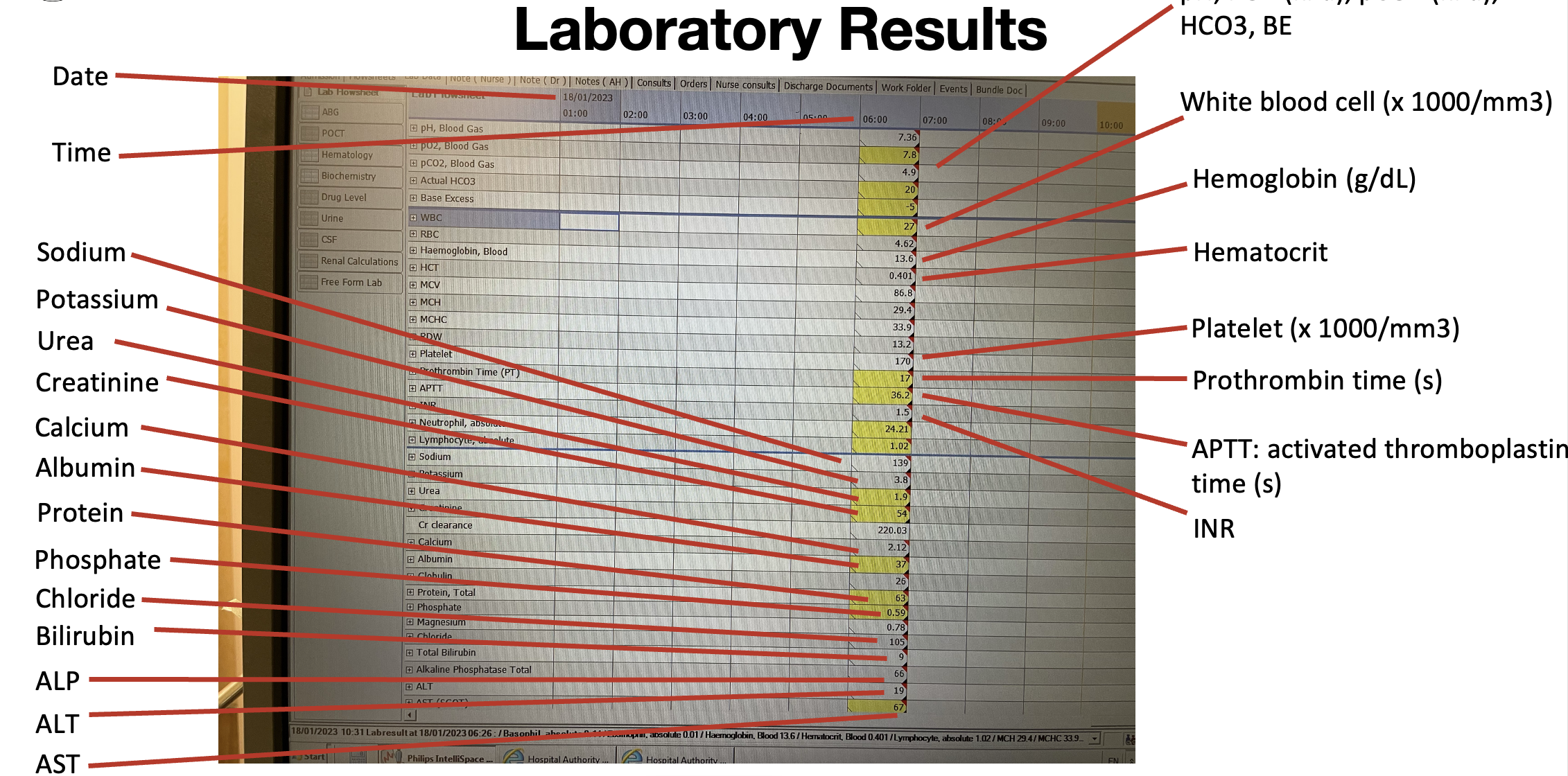


Panel B, Philips IntelliSpace in Hong Kong.


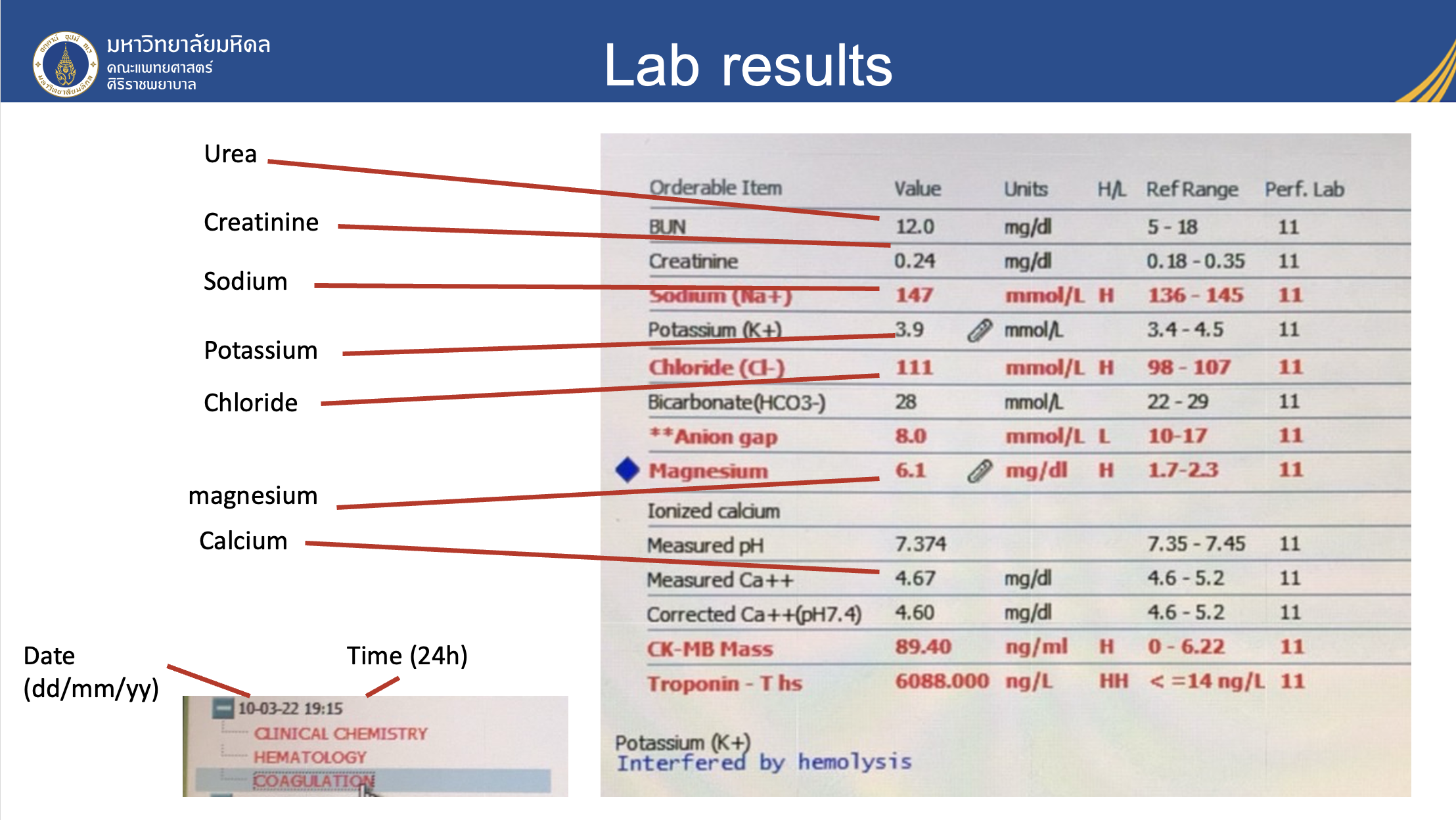


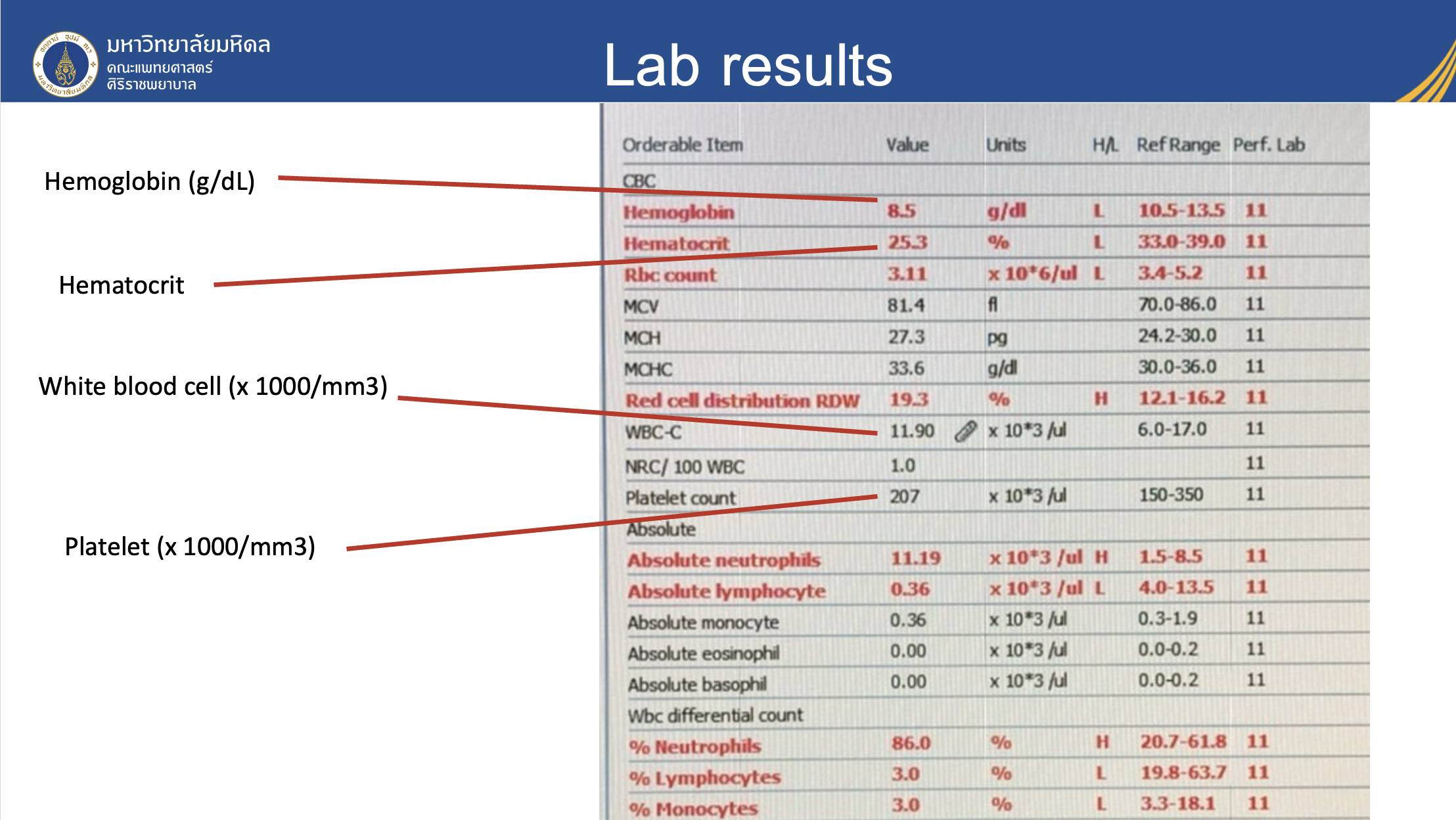


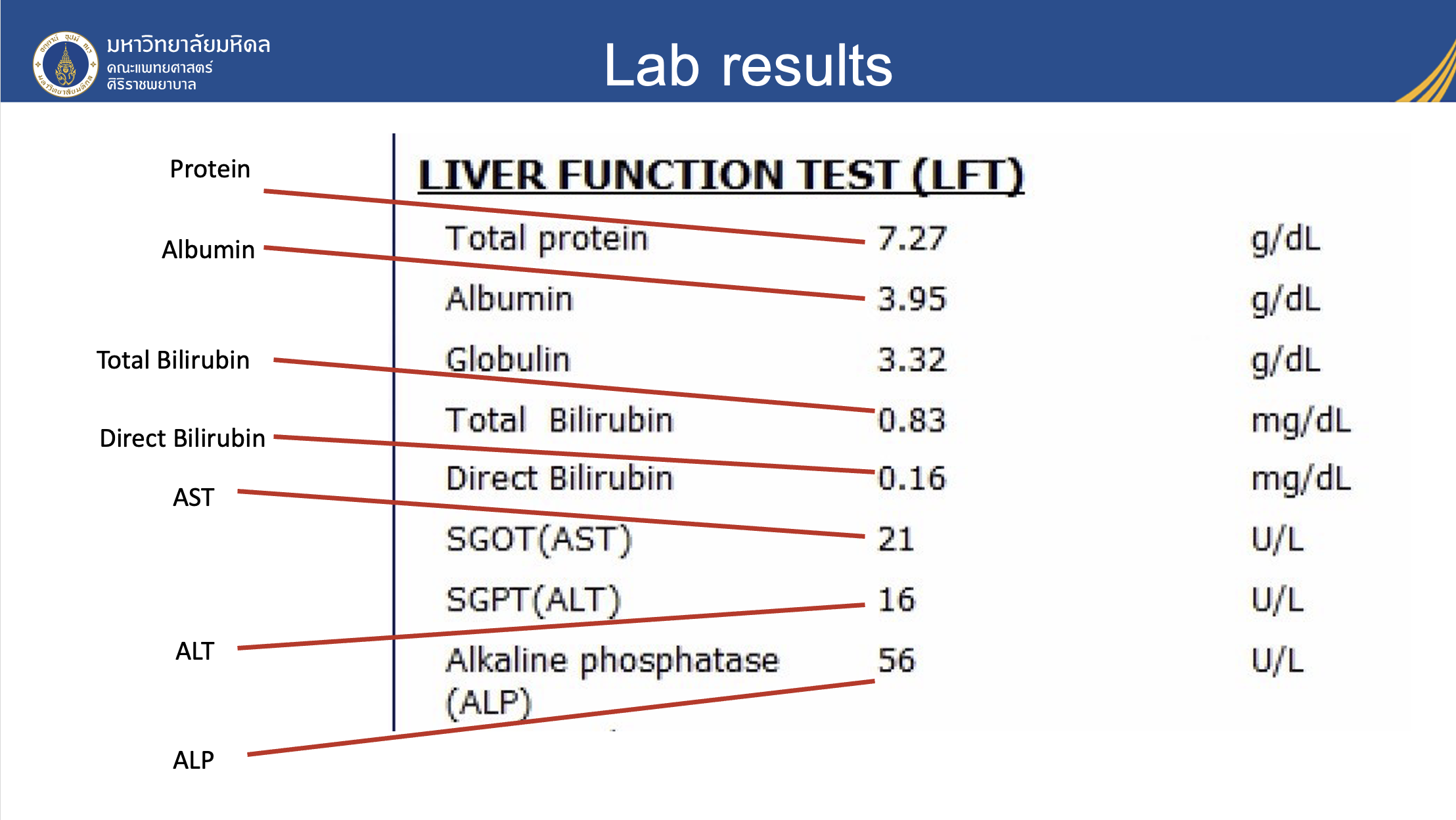


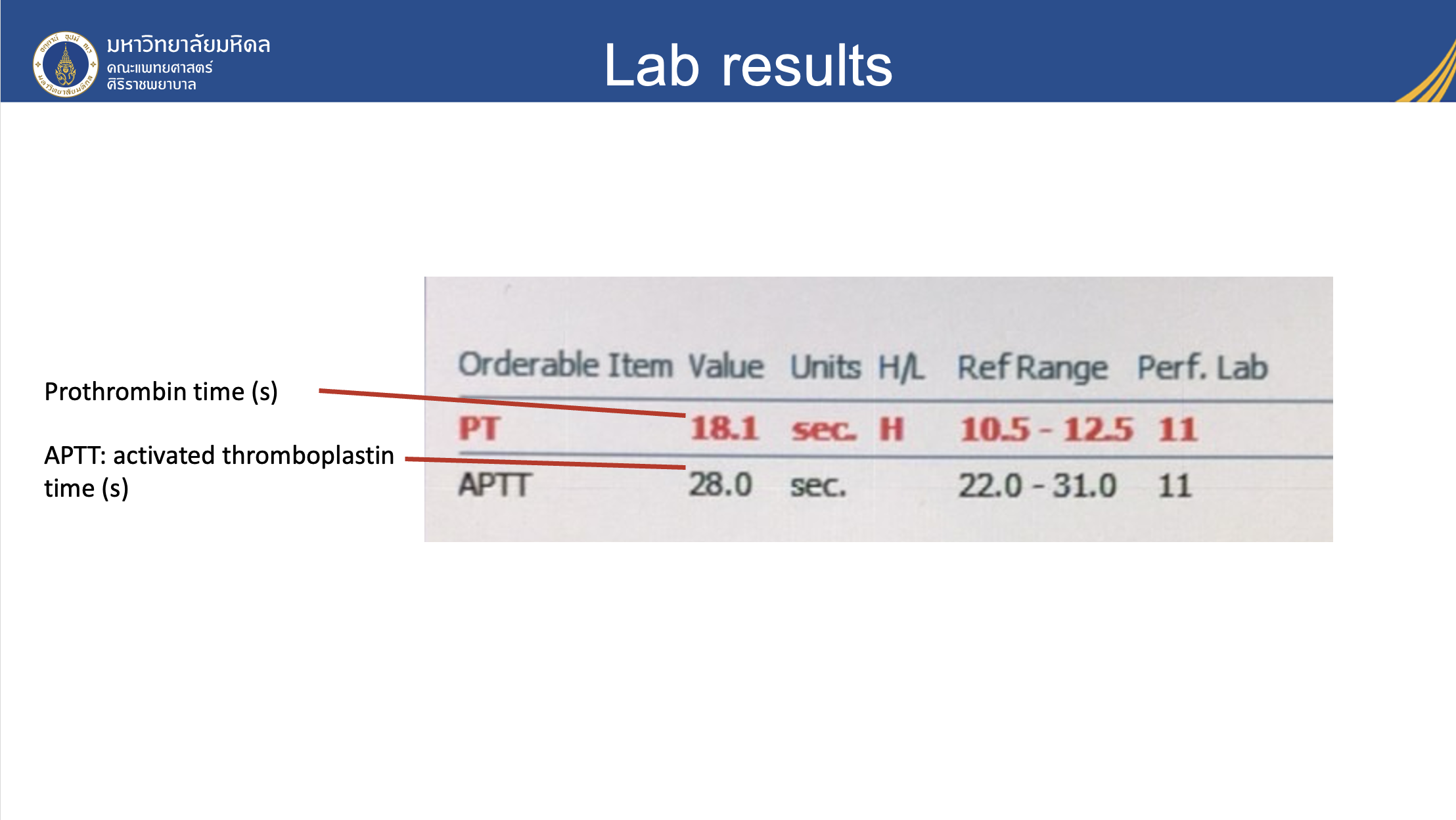


Panel C, Laboratory results in Thailand.
